# Development of introgression lines in high yielding, semi-dwarf genetic backgrounds to enable improvement of modern rice varieties for tolerance to multiple abiotic stresses free from undesirable linkage drag

**DOI:** 10.1038/s41598-020-70132-9

**Published:** 2020-08-04

**Authors:** Arvind Kumar, Nitika Sandhu, Challa Venkateshwarlu, Rahul Priyadarshi, Shailesh Yadav, Ratna Rani Majumder, Vikas Kumar Singh

**Affiliations:** 1 International Rice Research Institute, Metro Manila, Philippines; 2 Punjab Agricultural University, Ludhiana, India; 3 International Rice Research Institute, South Asia Hub, ICRISAT, Patancheru, Hyderabad India; 4 International Rice Research Institute, Guwahati, Assam India; 5 IRRI South Asia Regional Centre (ISARC), Varanasi, Uttar Pradesh India

**Keywords:** Genetics, Molecular biology, Plant sciences

## Abstract

Occurrence of multiple abiotic stresses in a single crop season has become more frequent than before. Most of the traditional donors possessing tolerance to abiotic stresses are tall, low-yielding with poor grain quality. To facilitate efficient use of complex polygenic traits in rice molecular breeding research, we undertook development of introgression lines in background of high-yielding, semi-dwarf varieties with good grain quality. The study reports the development and evaluations of over 25,000 introgression lines in eleven elite rice genetic backgrounds for improvement of yield under multiple abiotic-stresses such as drought, flood, high/low temperature. The developed introgression lines within each genetic background are near isogenic/recombinant inbred lines to their recipient recurrent parent with 50 to 98% background recovery and additionally carry QTLs/genes for abiotic stresses. The multiple-stress tolerant pyramided breeding lines combining high yield under normal situation and good yield under moderate to severe reproductive-stage drought, semi-dwarf plant type with good grain quality traits have been developed. The introgression lines in dwarf backgrounds open new opportunity to improve other varieties without any linkage drag as well as facilitate cloning of QTLs, identification and functional characterization of candidate genes, mechanisms associated with targeted QTLs and the genetic networks underlying complex polygenic traits.

## Introduction

The worldwide rice production is now facing the problem of a rapidly changing climate manifested as increased frequency and intensity of occurrence of multiple abiotic stresses. Climatic factors, such as drought, flood and extreme temperatures (low temperature and heat) are the main detrimental abiotic environmental stresses restricting plant growth and crop productivity^1^. The environmental stresses affect ~ 30% of 700 million poor people living in rainfed rice growing regions of Asia. Mega rice varieties are highly susceptible to these abiotic stresses, thereby suffering high yield losses. There is an urgent need to develop climate-change ready rice exploiting recent advances in genomics. The important goal of molecular quantitative genetics is to identify the genomic loci and specific alleles underlying the genetic variation in quantitative traits of interest. The recent advances in marker-assisted breeding have streamlined the food and feeder crops breeding programs^2,3^. In the past two decades, many quantitative trait loci (QTLs) associated with agronomic, physiological, grain quality, biotic and abiotic stress tolerance have been detected and some of them were cloned by using molecular markers^4–9^.

Before the advent of the genomics era in the early 2000s, many genes and QTLs governing abiotic stress tolerance were identified but they were not utilized successfully in breeding programs through marker assisted selection. Molecular marker-assisted introgression/QTL pyramiding has allowed fast and accurate selection and development of elite breeding lines with the target traits of interest. Despite the major advancements in modern era of genomics, moving from genomic region to the causal alleles remain a major challenge in genetics research. Pyramiding of favourable QTLs/genes present in traditional varieties provides a precise way to improve the varieties that excel in large number of characteristics^10–17^.

To improve grain yield, quality and biotic/abiotic resistance; development of introgression lines exploiting genomics-assisted breeding is mandatory. Developed introgression lines could be ideal and very useful material for QTL validation, fine mapping of QTL, gene discovery and in investigating the genetic and physiological mechanism in crop plants. Marker-assisted QTL introgression/pyramiding may be a suitable approach to get the desired phenotypic variance in improving grain yield under stress conditions in rice^18^. Near Isogenic lines provides an opportunity to validate the effect of single or few major effect QTLs in addition to an increase in the recovery of recurrent parent genome^19^. Utilizing the existing molecular markers for introgression/pyramiding of QTLs through marker-assisted selection is most desirable step in improving existing mega varieties and to develop new abiotic stress tolerance rice breeding lines/varieties. The introgression lines harboring desired QTLs/genes for multiple abiotic/biotic stresses in elite background with better performance may serve as valuable material for direct release to the farmer’s field. They may also serve as useful resource to study the epistatic interactions among introgressed QTLs and with the genetic background^17,20–22^.

Several major and consistent effect rice grain yield QTLs for reproductive stage drought stress; *qDTY*_*1.1*_ (on chromosome 1), *qDTY*_*2.1*_*, qDTY*_*2.2*_, *qDTY*_*2.3*_ (on chromosome 2), *qDTY*_*3.1,*_* qDTY*_*3.2*_ (on chromosome 3), *qDTY*_*4.1*_ (on chromosome 4), *qDTY*_*6.1*_*, qDTY*_*6.2*_ (on chromosome 6)*, qDTY*_*9.1*_ (on chromosome 9)*, qDTY*_*10.1*_ (on chromosome 10) and *qDTY*_*12.1*_ (on chromosome 12) have been identified to be present in traditional germplasm accession and their effect validated in different rice genetic backgrounds such as Swarna, IR64, MTU1010, TDK1-Sub1, MRQ74, MR219, Savitri and Vandana using different molecular marker technology^15,18,23–28^.

Submergence tolerance is controlled by a strong effect QTL *Sub1* from FR13A^29^, which can provide tolerance to rice up to 14 days of complete submergence. Flood tolerant version of many mega rice varieties such as Ciherang, Swarna, IR64 and TDK1 were developed using marker-assisted backcross breeding^30–32^.

The QTLs for low temperature tolerance at seedling, vegetative and booting stages^33–38^ along with the QTLs for tolerance to low temperature at reproductive stage^39–45^ have been identified in different populations. A total of 5 low temperature tolerance QTLs in a study with 122 RILs in F_13_ generation from a cross between Danteshwari and Dagaddeshi have been identified on chromosomes 1, 3, 6, 9 and 12^46^. The QTLs for heat tolerance in doubled haploid population^47^, recombinant inbred lines (RILs), back cross inbred lines (BILs) populations^48–57^ and chromosomal substitution lines^58^ have been reported.

Till date, many QTLs have been reported in different studies but very few have been deployed in marker-assisted breeding programs. Other than the stability of the QTLs effect across different genetic backgrounds and environments, one of the most important reason for this has been linkage drag of the identified QTLs with traits of less economic importance such as low grain yield, tall plant height, and poor grain quality^59^. The existing linkage drag need to be broken and QTLs need to be introgressed in backgrounds those possess other traits of economic importance. The aim of the study was to introgress newly identified QTLs to develop marker-assisted derived introgression lines in the background of popular cultivated varieties IR64, Swarna, MR219, TDK1, Samba Mahsuri, Kalinga 3, Vandana, Anjali and MTU1010 for tolerance to multiple abiotic stresses such as drought, low temperature and high temperature and make available appropriate genetic material to undertake more efficient, less cumbersome marker assisted introgression breeding programs as well as basic studies to clone genes, identify interactions and physiological mechanisms.

## Results

### Development of introgression lines

The drought tolerant introgression lines in background of a total of 11 popular lowland and upland adapted rice varieties free from the linkage drag with uneconomic traits were developed. The introgression schemes used to develop the dual drought-submergence tolerant introgression lines in background of lowland adapted rice varieties such as Swarna-Sub1, IR64-Sub1 and TDK1-Sub1 are summarised in Figs. S1, S2 and S3, respectively. The introgression schemes used to develop the drought tolerant introgression lines in background of lowland adapted rice varieties such as Savitri, Samba Mahsuri, MR219, MTU1010 and Swarna are summarised in Figs. S4, S5, S6, S7 and S8, respectively. The introgression schemes used to develop the introgression lines in background of upland adapted rice varieties such as Kalinga III, Vandana and Anjali are summarised in Figs. S9, S10, and S11, respectively. In addition, the multiple stress-tolerant; low temperature, heat and drought tolerant introgression lines in background of MTU1010 were developed.

The study generated a total of 25,000 introgression lines and evaluated 13,343 introgression lines under non-stress, 10,886 under reproductive stage drought stress, 400 under high and low temperature stress.

### Phenotypic characterization of introgression lines in different backgrounds

The phenotypic screening of the developed introgression lines were carried out across seasons, generations and environments. The significant phenotypic differences were observed among the recipient parents and introgression lines in all backgrounds under different conditions such as, reproductive stage drought stress, high and low temperature stress. The results of mean grain yield of parents and introgression lines and least significant difference (LSD_0.05_) of trial mean of introgression lines under non-stress (control), reproductive stage drought stress, high and low temperature stress are summarized in Table S1. The mean grain yield performance of introgression lines in comparison with the respective recurrent parent under non-stress and reproductive stage drought stress across seasons at IRRI, Philippines and SAH (Hyderabad, India) is presented in Figs. S1, S2, S3, S4, S5, S6, S7, S8, S9, S10, and S11.

### Phenotypic variability in multiple stress tolerant lowland adapted rice varieties

The phenotypic evaluations of a total of 9003 breeding lines in 58 experiments including 38 at IRRI, Philippines and 20 at IRRI, SAH were carried out between 2010–2017 (Table 1) in background of popular lowland adapted high-yielding varieties to validate the initial hypothesis of breakage of linkage drag and maintaining the effect of QTLs in the high yielding backgrounds. The mean grain yield of drought-submergence tolerant introgression lines in Swarna-Sub1 background varied from 3309 to 7878 kg ha^-1^ under non-stress and 634 to 2691 kg ha^-1^ under the reproductive stage drought stress (Table S1, Fig. S1). The variability for grain yield in IR64-Sub1 introgression lines ranged from 3024 to 7181 kg ha^-1^ under non-stress and 277 to 2998 kg ha^-1^ under the reproductive stage drought stress (Table S1, Fig. S2) whereas the mean grain yield in TDK1-Sub1 background ranged from 2715 to 6091 kg ha^-1^ and 198 to 1916 kg ha^-1^ under control non-stress and the drought stress, respectively (Table S1, Fig. S3). The variability for mean grain yield in low temperature-high temperature and drought tolerant introgression lines in MTU1010 background ranged from 5026 to 5176 kg ha^-1^ under non-stress, 1927 to 1985 kg ha^-1^ under the reproductive stage drought stress and 3585 to 3611 kg ha^-1^ under high temperature stress at reproductive stage (Table S1).

**Table 1:** The agronomic performance of selected lowland adapted promising breeding lines across different backgrounds under moderate stress (MS), severe reproductive stage drought stress (SS), non-stress (NS) and submergence (Sub) conditions.

Background	Introgression line	QTLs	(BCR) %	DTF (days)			PHT (cm)			GY (kg ha^-1^)			
				SS	MS	NS	SS	MS	NS	SS	MS	NS	Sub
Swarna-Sub1	IR 96322-34-223-B-1-1-1-1	*qDTY*_*1.1*_ + *qDTY*_*2.1*_ + *qDTY*_*3.1*_ + *Sub1*	94	98	95	95	66	71	85	1566	1893	6218	1371
	IR 96321-1447-651-B-1-1-2	*qDTY*_*1.1*_ + *qDTY*_*3.1*_ + *Sub1*	93	98	96	93	53	68	91	1525	1793	6474	1057
	IR 94391-131-358-19-B-1-1-1	*qDTY*_*3.1*_ + *Sub1*	98	96	95	92	64	70	88	2307	2570	7974	1650
	Swarna-Sub1	–	–	114	111	108	64	72	86	521	1088	5316	1118
	LSD_0.05_			12	9	7	11	10	10	585	676	1128	211
IR64-Sub1	IR 1022–42-16-1-1-2	*qDTY*_*2.2*_ + *qDTY*_*12.1*_ + *Sub1*	93	78	72	70	60	71	92	1426	1860	5	–
	IR 102793:1-11-229-3-1-1	*qDTY*_*4.1*_ + *qDTY*_*12.1*_ + *Sub1*	92	87	81	78	59	83	90	656	1900	5618	–
	IR 102793:1-11-64-1-1-2	*qDTY*_*1.2*_ + *qDTY*_*12.1*_ + *Sub1*	89	79	77	76	67	66	90	2002	2750	6145	–
	IR 102783:2-70-112-3-1-1	*qDTY*_*12.1*_ + *Sub1*	90	78	75	71	75	68	96	2210	3375	5143	–
	IR64	–	–	88	79	78	63	65	93	642	715	5002	–
	LSD_0.05_			10	9	6	11	9	10	848	998	421	–
TDK1-Sub1	IR 102777-5-83-1-2-7	*qDTY*_*6.1*_ + *qDTY*_*6.2*_	90	95	78	78	93	96	126	316	465	5590	–
	IR 102777-6-86-2-2-11	*qDTY*_*3.1*_ + *qDTY*_*6.1*_ + *qDTY*_*6.2*_	89	80	77	74	71	66	93	479	2625	5729	–
	IR 102777-6-86-2-2–14	*qDTY*_*6.2*_ + *Sub1*	90	93	77	76	71	70	105	422	1681	6629	–
	IR 102774-31-21-2-4-7	*qDTY*_*3.1*_ + *qDTY*_*6.1*_ + *Sub1*	90	90	78	75	93	73	118	434	3183	5610	–
	TDK1-Sub1	–	–	70	80	82	61	71	110	14	527	5412	–
	LSD_0.05_			7	3	1	18	5	13	305	929	882	–
Samba Mahsuri	IR 99734:1-33-304-1-5-8	*qDTY*_*2.2*_ + *qDTY*_*4.1*_	50	90	82	81	69	70	82	340	3015	5317	–
	IR 99734:1-33-304-1-5-10	*qDTY*_*2.2*_ + *qDTY*_*4.1*_	55	92	82	80	62	72	80	250	2448	5455	–
	IR 99734:1-33-69-1-12-8	*qDTY*_*2.2*_ + *qDTY*_*4.1*_	50	86	83	83	60	74	93	278	810	6525	–
	IR 99734:1-33-69-1-22-6	*qDTY*_*2.2*_ + *qDTY*_*4.1*_	65	95	85	84	62	73	89	307	1135	5195	–
	Samba Mahsuri	*-*	–	131	112	96	74	83	83	0	1269	5380	–
	LSD_0.05_			14	8	6	14	9	10	220	1017	823	–
MR219	IR 99784-255-78-2-3-1	*qDTY*_*2.2*_ + *qDTY*_*3.1*_ + *qDTY*_*12.1*_	73	80	77	79	65	64	78	1463	2615	5945	–
	IR 99784-156-87-2-4-1	*qDTY*_*3.1*_ + *qDTY*_*12.1*_	66	78	79	77	67	59	89	1085	2,932	6551	–
	MR219	*-*	–	107	109	97	63	62	99	44	573	5768	–
	LSD_0.05_			12	8	6	6	9	10	444	169	295	–
Savitri	IR 106,523-21-28-1-2-B	*qDTY*_*3.2*_	89	72	78	78	68	61	90	517	3205	5537	–
	IR 106529-20-40-3-1-B-B	*qDTY*_*3.2*_	88	74	77	76	68	64	88	343	2491	5696	–
	Savitri	*-*	–	113	96	103	48	66	104	22	903	4750	–
	LSD_0.05_			9	3	2	6	4	7	256	522	776	–
MTU1010	IR 91633-29-2-1-1-1-B	*qDTY*_*3.1*_ + *qDTY*_*6.1*_ + *qDTY*_*6.2*_	–	115	95	94	63	69	114	318	1629	9072	–
	IR 91631-28-1-2-1-3-B	*qDTY*_*2.2*_	–	117	96	95	63	68	115	312	1347	7680	–
	IR 91633-45-2-1-1-1-B	*qDTY*_*6.1*_ + *qDTY*_*6.2*_	–	116	95	96	64	63	106	278	1375	7480	–
	MTU1010	-	–	115	96	95	66	65	109	244	1377	8080	–
	LSD_0.05_			12	2	2	9	8	3	133	234	957	–
Swarna	IR 91648-B-89-B-6-1	*qDTY*_*3.2*_	–	111	100	87	64	85	128	193	1523	8293	–
	IR 91648-B-89-B-9-1	*qDTY*_*3.2*_	–	110	100	94	68	85	129	495	1140	5828	–
	IR 91648-B-89-B-8-1-B	*qDTY*_*3.2*_	–	111	100	95	69	84	128	265	1355	7609	–
	IR 91648-B-89-B-12-1-B	*qDTY*_*3.2*_	–	110	101	94	67	82	127	195	1057	7609	–
	Swarna	-	–	–	–	101	41	70	98	0	532	6672	–
	LSD_0.05_			5	6	5	10	8	4	188	258	1107	–

BCR(%), Back ground recovery in per centage; DTF, days to 50% flowering; PHT, Plant height in cm; GY, Grain yield in kg ha^-1^.

### Phenotypic variability in drought tolerant lowland adapted rice varieties

The phenotypic and genotypic evaluations of total 9632 breeding lines in 75 experiments including 42 at IRRI, Philippines and 33 at IRRI, SAH were carried out between 2010–2017 (Table 1) to develop introgression lines in background of popular lowland adapted high-yielding varieties. The mean grain yield of Samba Mahsuri introgression lines varied from 2395 to 8133 kg ha^-1^ and 227 to 2731 kg ha^-1^ under non-stress and the reproductive stage drought stress respectively (Table S1, Fig. S4) and in Savitri background from 3428 to 6125 kg ha^-1^ and 293 to 800 kg ha^-1^ under non-stress and reproductive stage drought stress respectively (Table S1, Fig. S5). The mean grain yield of introgression lines in MR219 background ranged from 4585 to 8294 kg ha^-1^ under non-stress and 486 to 2683 kg ha^-1^ under reproductive stage drought stress (Table S1, Fig. S6). The variability for mean grain yield in MTU1010 background ranged from 4661 to 9698 kg ha^-1^ under non-stress and 244 to 2970 kg ha^-1^ under reproductive stage drought stress (Table S1, Fig. S7). The grain yield variability in Moroberekan/3*Swarna introgression lines ranged from 4359 to 8918 kg ha^-1^ and 341 to 1648 kg ha^-1^ under control non-stress and reproductive stage drought stress, respectively (Table S1, Fig. S8).

### Phenotypic variability in drought tolerant upland adapted rice varieties

 The phenotypic evaluations of a total of 5684 breeding lines in 48 experiments including 33 at IRRI, Philippines and 15 at IRRI, SAH were carried out between 2010–2017 (Table 1) to develop introgression lines in background of upland adapted high-yielding rice varieties. The mean yield of Kalinga III introgression lines ranged from 2058 to 4790 kg ha^-1^ under non-stress and from 221 to 3253 kg ha^-1^ under reproductive stage drought stress (Table S1, Fig. S9). The mean grain yield of Vandana introgression varied from 2118 to 6072 kg ha^-1^ and 286 to 2408 kg ha^-1^ under non-stress and reproductive stage drought stress, respectively (Table S1, Fig. S10). The introgressed lines in Anjali background showed grain yield variability ranged from 2925 to 5121 kg ha^-1^ and 83 to 2141 kg ha^-1^ under control non-stress and reproductive stage drought stress, respectively (Table S1, Fig. S11).

### Genotypic characterization of introgression lines in different backgrounds

The genotypic selection (foreground and background selection) across generation advancements in all backgrounds were carried out to identify the high yielding stress tolerant promising lines with *qDTY* and *qDTY* combinations with high recurrent parent genome recovery (RPG) and free from linkage drag. The foreground/background selections were carried out and only the lines with desired phenotype and genotype (*qDTY*/*qDTY* combinations) were advanced to the next generation. The detailed information on the number of introgressed lines that were characterized genotypically in all the studied background in each season is presented in Table S1. The recurrent parent genome recovery (RPG) in the selected promising introgression lines carrying *qDTYs* in Swarna-Sub1 background ranged from 93 to 98%; 89 to 93% in IR64-Sub1 background; 89 to 90% in TDK-Sub1 background; 50 to 65% in Samba Mahsuri background; 88 to 89% in Savitri background and 66 to 73% in MR219 background (Table 1).

### Performance of lowland adapted promising introgression lines under different stresses

The introgression lines harboring *qDTY* and/or different *qDTY* combinations showed grain yield advantage under the reproductive stage drought stress, high temperature stress and low temperature stress with no/zero yield penalty under control/non-stress in all lowland adapted backgrounds, clearly signifying the breakage of linkage with tall plant height, low yield potential for QTLs possessing such linkage drag. The drought-submergence tolerant Swarna-Sub1 introgression lines possessing *qDTYs* showed grain yield advantage ranging from 1004 kg ha^-1^ (*qDTY*_*1.1*_ + *qDTY*_*3.1*_ + *Sub1*) to 1786 kg ha^-1^ (*qDTY*_*3.1*_ + *Sub1*) under severe stress, 705 (*qDTY*_*1.1*_ + *qDTY*_*3.1*_ + *Sub1*) to 1482 kg ha^-1^ (*qDTY*_*3.1*_ + *Sub1*) under moderate stress, 902 (*qDTY*_*1.1*_ + *qDTY*_*2.1*_ + *qDTY*_*3.1*_ + *Sub1*) to 2658 kg ha^-1^ (*qDTY*_*3.1*_ + *Sub1*) under non-stress and 253 kg ha^-1^ (*qDTY*_*1.1*_ + *qDTY*_*2.1*_ + *qDTY*_*3.1*_ + *Sub1*) to 532 kg ha^-1^ (*qDTY*_*3.1*_ + *Sub1*) under submergence conditions over Swarna-Sub1 (Table 1, Fig. S1). Most of the Swarna-Sub1 introgression lines showed recurrent parent recovery more than 90%. A reduction of 71 to 76% and 68 to 72% in grain yield was observed in Swarna-Sub1 introgression lines under severe stress and moderate stress, respectively compared to non-stress. The PHT (plant height) was reduced by 19 to 38 cm and 14 to 23 cm under severe and moderate stresses, respectively and DTF (days to 50% flowering) increased by 3 to 4 days. In IR64-Sub1 introgression lines, the grain yield reduction under severe and moderate stresses ranged from 57 to 88% and 34 to 66% respectively compared to non-stress (Table 1). Under severe stress, DTF difference was up to 9 days and PHT difference was up to 32 cm. The grain yield improvement over the recurrent parent (IR64) ranged from 14 (*qDTY*_*4.1*_ + *qDTY*_*12.1*_) to 1568 kg ha^-1^ (*qDTY*_*12.1*_) under severe stress, 1145 (*qDTY*_*2.2*_ + *qDTY*_*12.1*_) to 2660 kg ha^-1^ (*qDTY*_*12.1*_) under moderate stress and 141 (*qDTY*_*12.1*_) to 1143 kg ha^-1^ (*qDTY*_*1.2*_ + *qDTY*_*12.1*_) under non-stress (Table 1, Fig. S2). The recurrent parent genome recovery ranged from 89 to 93% (Table 1, Fig. S2).

The grain yield advantage of introgression lines in TDK1-Sub1 background varied from 302 kg ha^-1^ (*qDTY*_*6.1*_ + *qDTY*_*6.2*_) to 465 kg ha^-1^ (*qDTY*_*3.1*_ + *qDTY*_*6.1*_ + *qDTY*_*6.2*_) under severe stress, 938 (*qDTY*_*6.1*_ + *qDTY*_*6.2*_) to 2656 kg ha^-1^ (*qDTY*_*3.1*_ + *qDTY*_*6.1*_ + *Sub1*) under moderate stress and 178 kg ha^-1^ (*qDTY*_*6.1*_ + *qDTY*_*6.2*_) to 317 kg ha^-1^ (*qDTY*_*3.1*_ + *qDTY*_*6.1*_ + *qDTY*_*6.2*_) under non-stress condition compared to the recurrent parent (Table 1, Fig. S3). The background recovery was around 90%.

The grain yield improvement of introgression lines harboring *qDTY*_*2.2*_ + *qDTY*_*4.1*_ in Samba Mahsuri background ranged from 250 to 340 kg ha^-1^, 166 to 1746 kg ha^-1^ and 115 to 1145 kg ha^-1^ under severe, moderate and non-stress conditions, respectively over Samba Mahsuri (Table 1, Fig. S4). Under severe stress, the introgression line possessing *qDTY*_*2.2*_ + *qDTY*_*3.1*_ + *qDTY*_*12.1*_ in MR219 background showed higher grain yield improvement over MR219, whereas under moderate stress and non-stress the line possessing *qDTY*_*3.1*_ + *qDTY*_*12.1*_ showed higher grain yield improvement (Table 1, Fig. S6). The large reduction in grain yield under severe stress compared to non-stress was observed in the introgression lines in TDK1-Sub1 (Fig. S3), Samba Mahsuri (Fig. S4), Savitri (Fig. S5), MTU1010 (Fig. S7) and Swarna (Fig. S8) backgrounds. Under moderate stress grain yield improvement of 252 kg ha^-1^ (*qDTY*_*3.1*_ + *qDTY*_*6.1*_ + *qDTY*_*6.2*_) over MTU1010 was observed in the drought tolerant introgression lines in MTU1010 background. The grain yield advantage under severe drought stress, moderate drought stress and control non-stress conditions was 495, 991 and 1621 kg ha^-1^, respectively in Swarna^*^3/Moroberekan introgression lines (*qDTY*_*3.2*_) compared to the recurrent parent (Table 1, (Fig. S7).

The stringent phenotypic selection was employed to select multiple stress tolerance lines possessing tolerance to low temperature, high-temperature and drought stress. The introgression lines IR 128762-187-1-B-B-B-B, IR 128762-83-B-B-B-B-B and IR 128,762-155-2-B-B-B-B showed better performance over other introgression lines and parents (MTU1010 and IR 91648-B-117-B-1-1) under multiple stress conditions (Table 2). The variation in low temperature tolerance at germination and seedling stage was observed. IR 128762-187-1-B-B-B-B was found to be strongly low temperature tolerant at seedling stage and tolerant at germination stage, IR 128762-83-B-B-B-B-B was moderately low temperature tolerant at seedling stage and tolerant at germination stage and IR 128762-155-2-B-B-B-B was strongly low temperature tolerant at seedling stage and tolerant at germination stage (Table 2). The yield potential of selected lines under non-stress condition ranged from 5329 to 6617 kg ha^-1^, under RS from 2111 to 2571 kg ha^-1^ and under high-temperature stress from 3694 to 4394 kg ha^-1^ (Table 2). The percentage reduction in grain yield (61%) under reproductive stage drought stress was more as compared to the yield reduction under reproductive stage high-temperature stress (34%) (Table 2).

**Table 2:** The performance of selected multiple stresses tolerant promising lines under seedling and germination stage Low temperature stress, reproductive stage drought stress (SS), non-stress (NS) and reproductive stage high temperature stress (RS_HT).

S. no	Introgression line	Low temperature tolerance score		Grain yield (kg ha^-1^)			% Yield reduction		RS_HT
		Seedling stage^a^	Germination stage^b^	NS	RS	RS_HT	RS compared to NS	RS_HT compared to NS	Spikelet^a^ fertility
1	IR 128762-187-1-B-B-B-B	1	1	6617	2571	4394	61.2	33.6	3
2	IR 128762-83-B-B-B-B-B	4	1	5906	2339	4174	60.4	29.3	3
3	IR 128762-155-2-B-B-B-B	1	1	5566	2209	3920	60.3	29.6	3
4	IR 128762-179-1-B-B-B-B	1	1	5495	2181	3828	60.3	30.3	3
5	IR 128762-352-B-B-B-B-B	9	2	5462	2163	3694	60.4	32.3	3
6	IR 128762-187-4-B-B-B-B	3	1	5329	2111	3888	60.4	27.0	3
7	IR 128762-4-2-B-B-B-B	1	1	5418	2146	3881	60.4	28.4	3
8	MTU1010	7	2	5062	558	2184	89.0	56.9	7
9	IR 91648-B-117-B-1-1	1	1	5500	2178	3828	60.4	30.4	3
10	IR 133830-1-B-B-B-B*	1	1	5163	1515	3221	70.7	37.6	3
11	IR 64	7	2	5418	816	2044	84.9	62.3	7
12	Sahbhagi dhan	9	2	3775	2095	2814	44.5	25.4	3
	HSD_0.05_			996	471	595	–	–	–

^a^*Source*: Standard Evaluation System for Rice, International Rice Research Institute (SES, IRRI), 2014.

^b^*Source*: Han et al. 2006.

*Aromatic breeding line in background of Sahbhagi dhan.

### Performance of upland adapted promising introgression lines under drought stress

The introgression lines harboring *qDTY* and/or different *qDTYs* combinations with on par improvement in grain yield under reproductive stage drought stress with no yield penalty under non-stress were selected in all upland adapted backgrounds. The percent grain yield reduction under SS ranged from 79 to 84% and under MS from 71 to 79% compared to NS in Kalinga III background (Table 3, Fig. S9). The drought tolerant introgression lines possessing *qDTY*_*12.1*_ in Vandana background showed grain yield improvement of 578 kg ha^-1^, 1394 kg ha^-1^ and 208 kg ha^-1^ over Vandana under SS, MS and NS respectively (Table 3, Fig. S10). The PHT was reduced by 8 cm under SS. The grain yield improvement of introgression lines possessing *qDTY*_*12.1*_ + *qDTY*_*3.1*_ in Anjali background ranged from 516 to 613 kg ha^-1^ and from 156 to 540 kg ha^-1^ over the recurrent parent under SS and MS (Table 3, Fig. S11). The data on the DTF and PHT of the selected promising lines under different level of stresses is presented in Table 3.

**Table 3:** The agronomic traits performance of selected upland adapted promising breeding lines across different backgrounds under moderate stress (MS), severe reproductive stage drought stress (SS), non-stress (NS) and submergence (Sub) conditions.

Background	Promising NILs	QTLs	DTF (days)			PHT (cm)			GY (kg ha^-1^)		
			SS	MS	NS	SS	MS	NS	SS	MS	NS
Vandana	IR 84984-83-15-481-B	*qDTY*_*12.1*_	71	70	65	95	104	118	606	1635	4575
	Vandana	*qDTY*_*12.1*_	77	71	68	103	104	115	28	241	4368
	LSD_0.05_		5	4	3	7	6	4	354	321	389
Anjali	IR 99742:2-2-117-1-3-B	*qDTY*_*12.1*_ + *qDTY*_*3.1*_	71	65	61	71	95	104	1109	1665	4514
	IR 99742:2-11-17-1-9-B	*qDTY*_*12.1*_ + *qDTY*_*3.1*_	73	66	65	76	99	109	1147	1281	5149
	IR 97961-196-52-2-12-B	*qDTY*_*12.1*_ + *qDTY*_*3.1*_	73	66	64	78	103	112	1050	1533	4726
	IR 99742:2-2-124-1-1-B	*qDTY*_*12.1*_ + *qDTY*_*3.1*_	73	65	60	79	103	112	1133	1623	4
	Anjali	-	77	72	67	66	117	120	534	1125	4950
	LSD_0.05_		2	3	4	6	4	1	227	247	188
Kalinga III	IR 102614-59-B-1-1-B	*qDTY*_*12.1*_	71	66	59	102	125	137	869	1209	4236
	IR 102604-15-B-1-1-B	*qDTY*_*12.1*_	66	61	55	87	102	112	832	1212	4140
	IR 102612-31-B-1-1-B	*qDTY*_*12.1*_	74	58	72	99	106	113	648	844	4098
	IR 102613-67-B-1-1-B	*qDTY*_*12.1*_	56	53	50	80	112	119	729	911	4103
	Kalinga III	–	85	79	75	81	89	99	272	549	3732
	LSD_0.05_		5	4	5	6	6	4	321	285	222

DTF, days to 50% flowering; PHT, Plant height in cm; GY, Grain yield in kg ha^-1^.

### Genomic and environmental interactions

Despite being the large effect QTLs reported for the grain yield under drought stress at reproductive stage, the near isogenic lines with one, two, three or multiple QTLs showed grain yield variability in each of the genetic background. The contrasting performance of near isogenic lines possessing similar *qDTY* and also *qDTY* combinations may be due to the epistatic interactions of the introgressed QTLs among themselves and with the background marker loci under variable stresses level. The digenic interactions between marker loci and with the background were detected in low yielding nearly isogenic lines in IR64-Sub1, TDK1-Sub1, Savitri, MR219 and Samba Mahsuri backgrounds^17,22^. An epistatic interaction of *qDTY*_*12.1*_ with *qDTY*_*3.2*_ and *qDTY*_*2.3*_ in Vandana/Way Rarem background which was significantly improving yield of *qDTY*_*12.1*_ introgressed lines^60^. An epistatic interaction of *qDTY*_*4.1*_ on chromosome 4 and *qDTY*_*9.1*_ locus chromosome 9 with *qDTY*_*7.1*_ chromosome 7 in Samba Mahsuri background^17^^,^* qDTY*_*2.2*_ and *qDTY*_*3.1*_ with *qDTY*_*12.1*_ in MR219 background showed significant yield improvement over the lines having single QTLs^14^. The *qDTY*_*1.1*_ showed positive interactions with *qDTY*_*2.1*_, *qDTY*_*2.2*_, and *qDTY*_*3.1*_ across different genetic backgrounds used in the present study. Further, the positive interaction of *qDTY*_*2.2*_ with *qDTY*_*4.1*_, *qDTY*_*3.1*_ and *qDTY*_*12.1*_, *qDTY*_*3.1*_ with *qDTY*_*1.1*_, *qDTY*_*2.2*_, *qDTY*_*12.1*_, *qDTY*_*6.1*_, and *qDTY*_*6.2*_ also showed yield improvement over the lines with other *qDTYs* combinations in at least the genetic background used in the present study. Further, the release of different introgression lines with same or different QTL combinations indicated the role of QTL x environment interactions.

### Marker-assisted breeding product development

A total of 38 improved introgression lines in Swarna-Sub1 background, 6 in IR64-Sub1 background, 27 in Samba Mahsuri background, 6 in TDK1-Sub1 background, 20 in MR219 background, 7 in Savitri background, 51 in MTU1010 background, 7 in Swarna background, 3 in Sahbhagi dhan background were selected for lowland conditions. Further, a total 6 improved introgression lines in Vandana background, 10 in Anjali background and 25 in Kalinga III background were selected for upland conditions.

The developed near isogenic lines in the background of mega varieties not only have multiple QTLs but possess high grain and cooking quality traits, to the extent that some of the near isogenic lines have been released as varieties in different countries. The dual drought-submergence tolerance introgression lines in Swarna-Sub1 background, IR 96322-34-223-B-1–1-1–1 possessing *qDTY*_*1.1*_ + *qDTY*_*2.1*_ + *qDTY*_*3.1*_ + *Sub1* was released as variety under name CR dhan 801 in India in 2017; IR 96321-1447-651-B-1–1-2 possessing *qDTY*_*1.1*_ + *qDTY*_*3.1*_ + *Sub1* and IR 94391-131-358-19-B-1-1-1 possessing *qDTY*_*3.1*_ + *Sub1* were released as varieties in Nepal in 2017 under names Bahuguni dhan-1′ and ‘Bahuguni dhan-2′ respectively. The drought tolerant introgression line in IR64 background; IR 87707-445-B-B-B has been released in India for rainfed lowland regions as DRR Dhan 42; IR 87707-446-B-B-B in Nepal as Sukha dhan 4 in 2014 and in Myanmar as Yaenelo 4 in 2015. In addition, IR 87705-44-4-B-B, IR 87707-182-B-B-B and IR 87705-83-12-B-B have been released under names Yaenelo 5, Yaenelo 6 and Yaenelo 7, respectively in Myanmar in 2016. The release of marker-assisted breeding product in different countries prove the reasoning that QTLs for abiotic stress can be combined with high grain yield and good grain and cooking quality and also that the undesirable linkages reported earlier have been successfully broken to remove the linkage drag.

**Figure 1:**
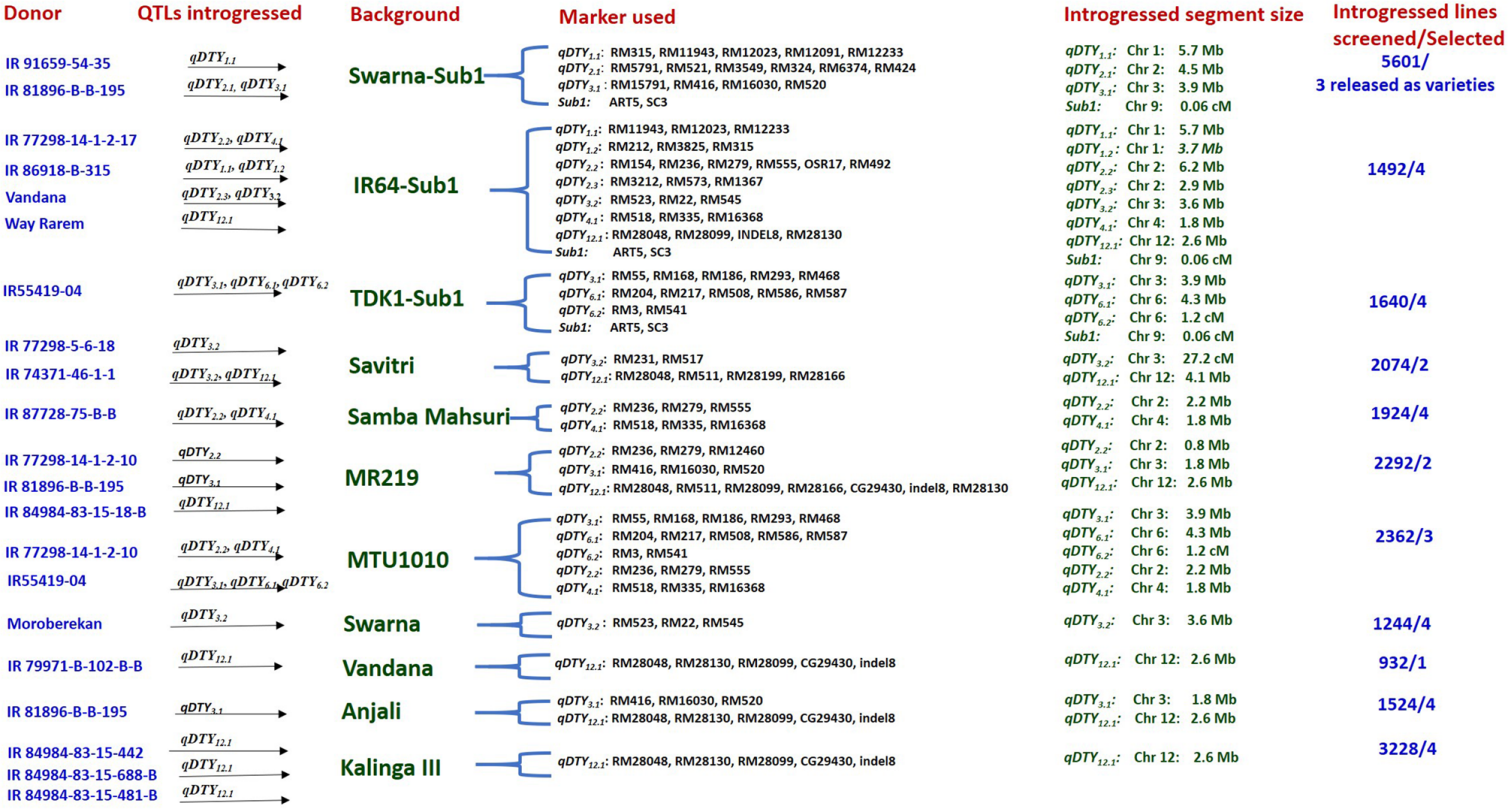
The summarized information on donors, QTLs used in marker-assisted introgression program, recipients, markers used for foreground and recombinant selection of introgressed region, size of introgressed segments and details on the number of total lines screened across different seasons/years and conditions and number of selected promising introgression lines in each background.

The summarized information on donors, QTLs used in marker-assisted introgression program, recipients, markers used for foreground and recombinant selection of introgressed region, size of introgressed segments and details on the number of total lines screened across different seasons/years and conditions and number of selected promising introgression lines in each background are presented in Fig. 1.

## Discussion

Development of introgression lines have been extensively employed in the identification and validation of the useful effects of genomic regions from the donor parents. Majority of the QTLs that have been detected so far in many studies^20,61–72^ and very few of them have been exploited and validated further in breeding programs. Some of the candidate genes that have already been cloned^73^ could be exploited in future breeding programs.

The genetic backgrounds such as Swarna, Swarna-Sub1, IR64, Samba Mahsuri, Vandana, Anjali, Kalinga III, MTU1010, Savitri, MR219 and TDK1-Sub1 that were used in this study to develop introgression lines are widely cultivated and accepted by the farmer in South and South-East Asian countries such as India, Nepal, Malaysia and Lao PDR. All these have production bottleneck due to their susceptibility towards drought, heat and low temperature stresses. Rice breeding at IRRI has made a significant progress in finding the key genomic regions associated with drought as well as submergence tolerance. Majority of the large-effect grain yield drought QTLs and *Sub1* for submergence have shown consistency in performances and explained a large part of phenotypic variances^18,23–25,29,30^.

The loci contributing grain yield improvement under drought stress reported to be collocated with the loci governing early days to flowering, plant height and reduced yield under irrigated conditions. The large segregating populations have been developed in Swarna-Sub1 and IR64 backgrounds to break these linkages and semi-dwarf medium duration lines have been successfully developed^74^. Apart from this, multilocational evaluations of the promising breeding lines offer opportunities to understand the QTL x environment interactions.

Combining multiple stress tolerances in single background of high yielding breeding line/variety are the breeder’s goals that have been targeted for a long time in the past. This is primarily because, in the years with well-characterised climate, the multiple stress-tolerant varieties should provide yield comparable to the popular high-yielding varieties in that area but shall also make the introgression of multiple lines easy, effective and without any undesirable effect. Rice drought molecular breeding at IRRI has witnessed some of the successful example of marker assisted introgression of drought QTLs in the elite backgrounds^14,15,17,18^.

The developed multiple stress tolerant introgression lines offer opportunities to the rice community to dissect the genetic variations existing among the introgression lines and to identify potentially useful pre-breeding lines to be serve as donors. In addition, very limited efforts have been performed to identify the QTL × background interactions influencing the overall expression of the target trait. There is a strong need to find such kind of unknown background noises for the effective deployment of the QTLs in marker-assisted breeding program. The developed introgression lines in different genetic backgrounds in the present study would be an important genetic material to predict such genetic interactions which complicates the genotype–phenotype relationship of complex quantitative traits^75,76^ such as drought, low temperature and high temperature tolerance. The developed introgression lines in different genetic backgrounds with same QTLs combination showed contrasting yield under different level of drought stress. The developed introgression lines in background of TDK1 Sub1, Samba Mahsuri, Savitri, MR219, MTU1010, Vandana, Anjali and Kalinga III can further be used for the genetic dissection of epistatic interactions contributing to the grain yield variability in rice under drought.

The complexity of genetic control of complex traits lead to wide variations in the performance of introgression lines. It has been observed that the same QTL showed variable performance across different backgrounds and environments. For a successful marker-assisted introgression program, it is very important to choose QTLs with large genetic effect and stability across wide range of environmental conditions and drought intensities^25,77^. Selection of the lines with higher yield based on phenotypic selection provided an opportunity to develop lines with positive interactions.

The marker-assisted breeding programme involving introgression of at least 2–3 major QTLs in different genetic backgrounds has permitted the development of many high-yielding multiple stress-tolerant lines. The introgression of 2–3 major QTLs in the background of widely cultivated popular high-yielding rice varieties lead an economic grain yield improvement of 1.0–1.2 t ha^–1^ under severe drought stress in farmers’ fields. The yield superiority of multiple stress tolerant lines across backgrounds and over generations clearly indicated that the tolerance of drought, submergence, low temperature and high temperature can be successfully combined even though these traits are controlled by different physiological mechanisms. The introgression lines possessed better grain quality than the traditional donors and so the development of multiple stress tolerant variety now with high quality has never been as easy as now. The development and release of marker-assisted breeding product for the rainfed lowland areas in IR64 and Swarna-Sub1^78^ backgrounds are the successful examples that should instigate the rice breeders to deploy the identified QTLs in the marker-assisted breeding programs targeting grain yield improvement under multiple abiotic stresses.

## Conclusions

The near isogenic lines with one, two, three and more QTLs have been developed in -eleven different elite backgrounds by the integration of phenotypic-genotypic selection approach. These near isogenic lines are free from linkage drag and hence we are able to combine increased yield under drought with high yield potential, dwarf plant type and good grain quality. Development of high-yielding and multiple stress tolerant introgression lines in wide range of backgrounds and for two diverse ecosystems validate the effectiveness of QTLs and marker-assisted introgression program. Development of such multiple stress tolerant rice varieties will provide opportunities to use these introgressed lines across different rice ecosystems to reduce yield under abiotic stresses and have an impact on increase in rice production and overall increase in the farm output in stress prone areas of South and South East Asia. The near isogenic lines with combination of QTLs brings a great advantage to breeding by providing opportunities to breeders to introgress QTLs in less time by just attempting one or two crosses as against three to four years that were spend earlier just to bring multiple QTLs together. The study clearly indicated that it is necessary to identify and break such linkage drags for effective use of QTLs in the trait development or breeding programs. The study also validated that once linkage drags are removed, it is possible to combine tolerance to multiple traits controlled by diverse physiological mechanisms—low temperature and high temperature; drought and low temperature, high yield and drought tolerance and low temperature. The findings from the current study can revolutionize the marker assisted breeding as very few of the large number of QTLs identified have been used in the past. The developed introgression lines in different genetic backgrounds are unique set of germplasm to the interaction studies to break the grain yield improvement barrier under multiple stresses. In addition, these lines may serve as an important material to study the functional characterization of candidate genes and genetic networks utilizing advances in functional genomics.

## Materials and methods

The present study was conducted at International Rice Research Institute (IRRI), Los Baños, Laguna (Philippines) and IRRI South Asia breeding hub (SAH), Hyderabad, India during years from 2012 to 2017.

### Breeding for drought tolerance at IRRI, Philippines

#### Plant material

The first step of breeding program is to identify the appropriate donor exploiting the genetic variations in the existing germplasm. To avoid undesirable linkages associated with the traditional donors, improved breeding lines with genomic regions associated with different abiotic stresses were used in the present study^79^. The improved breeding lines used as donors were IR 91659-54-35 (*qDTY*_*1.1*_), IR 77298-14-1-2-10 (*qDTY*_*2.2*_), IR 81896-B-B-195 (*qDTY*_*3.1*_), IR 77298-5-6-18 (*qDTY*_*3.2*_), IR 74371-46-1-1 and IR 84984-83-15-18-B (*qDTY*_*12.1*_), IR 86918-B-315 (*qDTY*_*1.1*_ + *qDTY*_*1.2*_), IR 81896-B-B-195 (*qDTY*_*2.1*_ + *qDTY*_*3.1*_), IR 87728-75-B-B and IR 77298-14-1-2-17 (*qDTY*_*2.2*_ + *qDTY*_*4.1*_), Vandana (*qDTY*_*2.3*_ + *qDTY*_3.2_), and IR55419-04 (*qDTY*_*3.1*_ + *qDTY*_*6.1*_ + *qDTY*_*6.2*_). The popular lowland adapted high-yielding varieties such as Swarna, Swarna-Sub1, IR64-Sub1, Samba Mahsuri, TDK1, MR219, Savitri and MTU1010, and the upland adapted rice varieties Kalinga 3, Vandana and Anjali were used as recipient parent to develop marker-assisted derived breeding lines for future climate-change ready varietal development.

#### Development of introgression lines

The Swarna-Sub1 introgression lines were developed using donors IR 91659-54-35 possessing *qDTY*_*1.1*_; IR 81896-B-B-195 possessing *qDTY*_*2.1*_ and *qDTY*_*3.1*_. The introgression lines in IR64-Sub1 background were developed from crossing involving donors IR 77298-14-1-2-17 possessing two *qDTYs* (*qDTY*_*2.2*_ and *qDTY*_*4.1*_)^18^^,^ IR 86918-B-315 possessing two *qDTYs* (*qDTY*_*1.1*_ and *qDTY*_*1.2*_), Vandana possessing two *qDTYs* (*qDTY*_*2.3*_ and *qDTY*_3.2_) and Way Rarem possessing one *qDTY* (*qDTY*_*12.1*_). The introgression lines in TDK1-Sub1 background were developed using BC_1_F_3:5_ derived pyramided lines from the cross IR55419-04/2*TDK1^15^. Savitri introgression lines were developed involving the pyramided lines derived from IR 77298-5-6-18/2*Savitri//IR 74371-1-1/2*Savitri///2*Savitri carrying *qDTY*_*3.2*_ and *qDTY*_*12.1*_ QTLs in Savitri background^15,26,27^. The introgression lines in Samba Mahsuri background were developed involving IR 87728-75-B-B*,* a donor of *qDTY*_*2.2*_ and *qDTY*_*4.1*_^17^. MR219 introgression lines were generated using three donors IR 77298-14-1-2-10 (*qDTY*_*2.2*_), IR 81896-B-B-195 (*qDTY*_*3.1*_) and IR 84984-83-15-18-B (*qDTY*_*12.1*_)^14^. The introgression lines in MTU1010 background were developed using IR 77298-14-1-2-10 (*qDTY*_*2.2*_) and IR 55419-04 (*qDTY*_*3.1*_, *qDTY*_*6.1*_ and *qDTY*_*6.2*_). The Moroberekan/3*Swarna introgression lines were developed using Moroberekan as donor (*qDTY*_*3.2*_). The introgression lines in Vandana background was developed using IR 79971-B-102-B-B (*qDTY*_*12.1*_) as donor parent, in Anjali background were developed involving donors for *qDTY*_*3.1*_ (IR 81896-B-B-195-2), and *qDTY*_*12.1*_ (IR 84984-83-15-18-B-B-93) and in Kalinga III background using different donors of *qDTY*_*12.1*_ (IR 8498483-15-442, IR 84984-83-15-688-B and IR 84984-83-15-481-B).

#### Phenotyping screening

The screening of marker-assisted derived breeding lines was conducted under lowland and/or upland reproductive stage drought stress (RS) and irrigated control/non-stress (NS) conditions at IRRI (International Rice Research Institute). The experiments were planted in two to three replications in single or two or four-rows plots with row length of 5 m and 2.0 to 3.0 m in lowland and upland condition, respectively in an alpha lattice design. The lowland experiments involved transplanting that involved the transplantation of 21-days-old seedlings in the field with a single seedling per hill. The long duration introgression lines were planted 15–20 days earlier than the medium duration varieties. The field management activities of the lowland experiments were carried out in similar way as described earlier by Venuprasad et al.^24^. The detailed information on number of entries tested, generation, experimental design, treatments and number of replications is presented in Table S1.

Upland experiments were established under dry direct seeded conditions by maintaining 20 cm × 20 cm between row to row and plant to plant distance. Field management practises under upland was done as described by Bernier et al.^23^. In brief, under upland conditions, combination of Oxadiazon (pre-emergence herbicide) at 6 days after seeding (DAS) @ 0.5 kg ai ha^-1^, Bispyribac sodium (early post emergence) at 11 and 22 DAS @ 0.03 kg ai ha^-1^ in addition to spot weeding at 35 and 55 DAS were used to control the weeds. IPM (Integrated pest management) practices was followed involving ditrac @ 0.05 g kg^-1^ (0.005%), brodifacoum as rat bait to control rats, and pre-seeding application of the 0.075 kg ai ha^-1^ of Fipronil along bunds and the plot edges at 7 DAS.

*Lowland drought screening.* At 30 days after transplanting (DAT), complete drainage of water was carried out and the drought stress at reproductive stage was initiated by holding irrigation. The initiation of reproductive stage drought stress for the long duration lines were adjusted based on their reproductive cycle. When ~ 75% of the population showed symptoms of severe leaf rolling and the water table depth remained > 100 cm for approximately > 2 weeks; a life-saving irrigation was applied through the flash flooding. After 24 h, complete drainage of the water from the field was carried out again to impose the second cycle of drought stress. The 1.1 m PVC (polyvinyl chloride) pipe was installed in the soil to measure the water table depth at regular intervals in the experimental fields. The depletion of the water table level was measured using a meter scale day-to-day after the onset of the drought stress.

*Upland drought screening.* The upland experiments were irrigated using sprinkler-irrigation up to 45 DAS, with application of irrigation twice a week during seedling establishment stage and in early vegetative developmental stage. Thereafter, stress at reproductive stage was initiated by holding irrigation. The plots were re-irrigated when the tensiometer at 30 cm soil depth showed reading below – 50 kPa and most of the lines showed wilting and leaf drying. This kind of cyclic stress is effective in drought screening of populations consisting of variable genotypes having broad range of growth duration^80^^,^ensuring all breeding lines receive an adequate level of stress during the reproductive stage of development. All the cultural practices were same in upland non-stress and reproductive stage stress experiments, except that in non-stress experiments the irrigation was continued two times a week up to 10 days before the harvest of the crop.

*Biotic stress screening.* The introgression lines at F_2_ and F_7_/F_8_ stages were also evaluated for the disease resistances such as blast which is caused by *Magnaporthe oryzae* and bacterial blight which is caused by *Xanthomonas oryzae* pv*. Oryzae*. The blast screening was done using the mixed inoculum for the races presents in the Philippines at 10 DAS in the blast nursery. The plants possessing genes viz*. pik-s, pi2, pi5(t)* and *pi9* showed resistance against the inoculum. The inoculation and screening for the bacterial blight race 1, *PXO61* and race 2, *PXO86* was done at maximum tillering stage in field following Kauffman^81^. The resistant or susceptibility of introgression lines against the blast and bacterial blight diseases was observed on IRRI, SES scale^82^.

#### Phenotypic data collection

The agronomic data on days to 50% flowering (DTF), plant height (PHT), and grain yield (GY) were recorded for all the experiments. DTF was recorded when ~ 50% the plants in a plot showed panicle exertion. At maturity, three random plants per plot were measured from the soil surface to the tip of the highest panicle on main tiller to record the PHT (cm). The plant samples at maturity were harvested, dried to 14% of the moisture before weighing, and weights to record the GY (kg ha^–1^). The developed introgression lines were screened under both reproductive-stage drought (RS) and irrigated control non-stress (NS) conditions along with the recipient parent, high-yielding check varieties and drought-tolerant donors. The imposed reproductive stage drought stress was classified into severe and moderate drought stress based on the relative reduction in the grain yield compared to non-stress^79^. The experiments with yield reduction of < 70% were characterize as severe stress (SS) and with yield reduction of 31–70% were categorized as moderate stress (MS). The selected introgression lines were also evaluated under national/state trials in different countries.

## IRRI SAH

### Breeding for drought, heat and low temperature tolerance

To develop multiple stress tolerance lines possessing tolerance to low temperature, high-temperature and drought stresses, a recombinant inbred lines (RILs) population consisting of 400 RILs involving IR 91648-B-117-B-1-1 (improved breeding lines from Swarna^*^3/Moroberekan)^83^ as donor parent and MTU1010 as recipient parent was developed. Pedigree based breeding was employed to develop multiple tolerance lines involving stringent phenotypic selection for the targeted traits. A series of experiments following standard agronomic practices were carried out between 2013 to 2017 at International Rice Research Institute-South Asia Hub (IRRI-SAH), Patancheru which is at 78° 16′ longitude and at 17° 32′ latitude. The experiments were planted in two replications in twelve-rows plots with 3 m row length maintaining 20 (plant to plant) × 15 (row to row) cm spacing under transplanted conditions in randomized complete block design (RCBD).

### Phenotypic screening and data collection

The phenotypic screening of breeding lines for the drought stress at reproductive stage was carried under natural conditions as above-mentioned procedure in both dry and wet seasons. The low temperature and high-temperature screening were carried based on the SES (Standard evaluation system for rice)^84^. Germination vigor and the seedling survival rate were two main criteria that were used for the evaluation of low temperature tolerance in rice at germination stage^85^. The high-temperature screening at reproductive stage and low temperature tolerance screening at germination stage was carried out under field condition in dry season while the low temperature tolerance screening at seedling stage was conducted under field in the dry season as well as under controlled condition in the wet seasons. To compare the performance of breeding lines under treatment and non-stress (control) conditions, non-stress experiments were carried out for each drought, heat and low temperature tolerance experiments. Three plants per introgression line were chosen for the measurement of all traits. The phenotypic data on DTF, PHT, number of productive tillers (counted manually), flag leaf length (FLL,cm, panicle length (PL,cm, number of filled grains per panicle (FG/P, spikelet fertility (SF; %, 1000-grain weight (1000 GW; g), grain yield (GY; kg ha^-1^) and grain type was recorded.

### Genotyping of the developed introgression lines in different backgrounds

In the marker-assisted introgression program, rice microsatellite (SSR; simple sequence repeats) markers were extensively used. Earlier reported SSR markers associated with the various grain yield *qDTYs*^15,17,18,23–28^ were used to detect the targeted region in the developed introgression lines in various backgrounds (Table S2).

The fresh-young leaves from the 21 days old seedlings were collected from each introgression line and the respective parents. The genomic DNA was extracted using the modified CTAB protocol^86^. Polymerase chain reaction (PCR) amplification was performed with each PCR reaction mixture of 15 μl containing DNA (10 ng), PCR buffer (1 ×), dNTPs (100 μM), oligonucleotide primers (100 μM) and Taq polymerase (1 unit). The PCR products were then resolved on high-resolution PAGE (polyacrylamide gel electrophoresis) (CBS scientific, MGV-202-33) ranged from 6–8% (v/v) depending on the SSR markers product size. The separated fragments of DNA were stained with the SYBER Safe™ before visualizing under UV trans-illuminator (AlphaImager™ System). The strong associations of the reported markers with the introgressed regions and the co-dominance nature of SSRs allowed the easy detection of heterozygotes. As many of the identified *qDTYs* regions that were used in the introgression program were not fine mapped, all SSRs present within the identified genomic regions/QTLs were used in the genotyping of the introgression lines to avoid a loss of the candidate genes associated with abiotic stresses tolerance (drought, low temperature and high temperature) in the region due to the crossover. To reduce the forward genotyping cost and labor, sequential genotyping approach was used. Firstly, the peak maker for each QTL was used to genotype the F_2_ segregants, then the breeding lines harbouring the respective donor allele at peak marker were further genotyped with the flanking markers, followed by genotyping with all the markers underlying the QTL regions.

### Background recovery

To study the background recovery of the selected introgression lines, background genotyping was carried out. A total of ~ 600 SSR markers equally distributed on all the 12 chromosomes (https://www.gramene.org/) were used to study the polymorphism between the parents. The selected polymorphic SSR markers; 156, 111, 101, 89, 80, and 102 SSR markers were used to estimate the background recovery percentage in Swarna, IR64, TDK1-Sub1, Savitri, Samba Mahsuri, MR219 backgrounds, respectively were chosen. Background study has not been conducted yet in Anjali and Kalinga backgrounds. The background recovery percentage (BC%) of the selected introgression lines in different backgrounds were calculated as Vishwakarma et al.^87^.

$${\text{Background}}\;{\text{recovery}}\;{\text{BC\% }} = \frac{{2\left( {\text{B}} \right) + \left( {\text{H}} \right)}}{2N} \times 100$$

where B is the SSR marker loci homozygous for the genetic background; H is the number of markers loci in heterozygous; N is the total number of polymorphic markers that were used for the background genotyping.

### Statistical analysis

The agronomic data collected from all the experiments were analysed statistically. The experiment means and SED (standard error of difference) were calculated using the statistical software *PBTools v1.4* developed at IRRI. The LSD (Least significant difference) at *P* = 5% and 1% significance were used to make the comparison of the test entries means and to estimate the significant differences of the traits of interest between the parents and the introgression lines.

The mixed linear model was used to calculate the analysis of variance:

$$Yijk = \mu + Gi + Rj + BK \left( {Rj} \right) + eijk$$

where µ is the overall mean, Gi is the effect of i^th^ genotype, Rj is the effect of j^th^ replicate, BK (Rj) is block effect of the j^th^ replicate and eijk is the error. Genotypes were kept as fixed and the replication and block effects were kept random for estimating entry means.

## Supplementary information

Supplementary Information.
