# Supplementary material for: Development of introgression lines in high yielding, semi-dwarf genetic backgrounds to enable improvement of modern rice varieties for tolerance to multiple abiotic stresses free from undesirable linkage drag
Source: Sci Rep. 2020 Aug 4;10:13073. doi: 10.1038/s41598-020-70132-9 (PMC7403580; doi:10.1038/s41598-020-70132-9)
Supplement: Supplementary file 1 — Supplementary Information. [file 41598_2020_70132_MOESM1_ESM.docx]

**Development of introgression lines in high yielding, semi-dwarf genetic backgrounds to enable improvement of modern rice varieties for tolerance to multiple abiotic stresses free from undesirable linkage drag**

Arvind Kumar*^1,5^, Nitika Sandhu^1,2^, Challa Venkateshwarlu^3^, Rahul Priyadarshi^3,4^, Shailesh Yadav^1^, Ratna Rani Majumder^1^, Vikas Kumar Singh^3^

^1^ International Rice Research Institute, Metro Manila, Philippines

^2^ Punjab Agricultural University, Ludhiana, India

^3^ International Rice Research Institute, South Asia Hub, ICRISAT, Patancheru, Hyderabad, India

^4^ International Rice Research Institute, Guwahati, Assam, India

^5^ IRRI South Asia Regional Centre (ISARC), Varanasi, Uttar Pradesh, India

*Corresponding author : [a.kumar@irri.org](mailto:a.kumar@irri.org)

Arvind Kumar : [a.kumar@irri.org](mailto:a.kumar@irri.org)

Nitika Sandhu : [nitikasandhu@pau.edu](mailto:nitikasandhu@pau.edu)

Challa Venkateshwarlu : [c.venkateshwarlu@irri.org](mailto:c.venkateshwarlu@irri.org)

Rahul Priyadarshi : [r.priyadarshi@irri.org](mailto:r.priyadarshi@irri.org)

Shailesh Yadav: : [shailesh.yadav@irri.org](mailto:shailesh.yadav@irri.org)

Ratna Rani Majumder : [r.majumder@irri.org](mailto:r.majumder@irri.org)

Vikas Kumar Singh : [v.k.singh@irri.org](mailto:v.k.singh@irri.org)

**Table S1.** Detailed information on experiments, number of entries tested, generation, experimental design, number of replications and treatments across locations.

| Sr No | Location | Environment | Background | Year/  Season | Trt | No of entries | Experimental design | Rep | Generation | Recipient Parent | Trial Mean | LSD_0.05_ | Genotypic Selection |
| --- | --- | --- | --- | --- | --- | --- | --- | --- | --- | --- | --- | --- | --- |
| 1 | IRRI_HQ | Lowland | Swarna-Sub1 | 2012DS | NS | 663 | Augmented RCBD | - | F_3_ | 3818 | 5077 | 450 | Foreground, Recurrent |
| 2 | IRRI_HQ | Lowland | Swarna-Sub1 | 2012DS | RS_DR | 366 | Augmented RCBD | - | F_3_ | 2203 | 2691 | 264 | Foreground, Recurrent |
| 3 | IRRI_HQ | Lowland | Swarna-Sub1 | 2012WS | NS | 754 | Augmented RCBD | - | F_4_ | 5146 | 4760 | 680 | Foreground, Recurrent |
| 4 | IRRI_HQ | Lowland | Swarna-Sub1 | 2013DS | NS | 432 | Augmented RCBD | - | F_5_ | 2106 | 3615 | 1188 | Foreground, Recurrent |
| 5 | IRRI_HQ | Lowland | Swarna-Sub1 | 2013DS | RS_DR | 432 | Augmented RCBD | - | F_5_ | 764 | 1083 | 2181 | Foreground, Recurrent |
| 6 | IRRI_HQ | Lowland | Swarna-Sub1 | 2014DS | NS | 426 | Augmented RCBD | - | F_7_ | 5818 | 7878 | 3010 | Foreground, Recurrent |
| 7 | IRRI_HQ | Lowland | Swarna-Sub1 | 2014DS | RS_DR | 426 | Augmented RCBD | - | F_7_ | 799 | 1652 | 1169 | Foreground, Recurrent |
| 8 | IRRI_HQ | Lowland | Swarna-Sub1 | 2014WS | NS | 48 | Alpha lattice (8x6) | 2 | F_8_ | 3976 | 3309 | 930 | Foreground, Recurrent |
| 9 | IRRI_HQ | Lowland | Swarna-Sub1 | 2015DS | NS | 35 | Alpha lattice (5x7) | 2 | F_8_ | 5904 | 6747 | 1129 | Foreground, Recurrent |
| 10 | IRRI_HQ | Lowland | Swarna-Sub1 | 2015DS | RS_DR | 35 | Alpha lattice (5x7) | 2 | F_8_ | 755 | 1510 | 586 | Foreground, Recurrent |
| 11 | IRRI_HQ | Lowland | Swarna-Sub1 | 2015WS | NS | 52 | RCBD | 2 | F_8_ | 5358 | 5222 | 781 | Foreground, Recurrent |
| 12 | IRRI_HQ | Lowland | Swarna-Sub1 | 2016DS | NS | 48 | Alpha lattice (4x4) | 2 | F_8_ | 5321 | 5129 | 446 | Foreground, Recurrent |
| 13 | IRRI_HQ | Lowland | Swarna-Sub1 | 2016DS | RS_DR | 48 | RCBD | 2 | F_8_ | 282 | 695 | 105 | Foreground, Recurrent |
| 14 | IRRI_HQ | Lowland | Swarna-Sub1 | 2017DS | NS | 6 | RCBD | 2 | F_8_ | 6156 | 6212 | 650 | Foreground, Recurrent |
| 15 | IRRI_HQ | Lowland | Swarna-Sub1 | 2017DS | RS_DR | 6 | RCBD | 2 | F_8_ | 1085 | 1647 | 554 | Foreground, Recurrent |
| 16 | IRRI_SAH | Lowland | Swarna-Sub1 | 2012WS | RS_DR | 258 | Augmented RCBD | - | F_4_ | 472 | 1093 | 1287 | Foreground, Recurrent |
| 17 | IRRI_SAH | Lowland | Swarna-Sub1 | 2013DS | NS | 336 | Augmented RCBD | - | F_6_ | 6363 | 6152 | 3426 | Foreground, Recurrent |
| 18 | IRRI_SAH | Lowland | Swarna-Sub1 | 2013WS | NS | 362 | Augmented RCBD | - | F_6_ | 7500 | 7448 | 2694 | Foreground, Recurrent |
| 19 | IRRI_SAH | Lowland | Swarna-Sub1 | 2013WS | RS_DR | 238 | Augmented RCBD | - | F_6_ | 2297 | 2289 | 531 | Foreground, Recurrent |
| 20 | IRRI_SAH | Lowland | Swarna-Sub1 | 2014DS | NS | 118 | RCBD | 2 | F_7_ | 5362 | 4522 | 1291 | Foreground, Recurrent |
| 21 | IRRI_SAH | Lowland | Swarna-Sub1 | 2014DS | RS_DR | 118 | RCBD | 2 | F_7_ | 276 | 1067 | 590 | Foreground, Recurrent |
| 22 | IRRI_SAH | Lowland | Swarna-Sub1 | 2014WS | NS | 45 | Alpha lattice (5x9) | 2 | F_8_ | 5883 | 6186 | 733 | Foreground, Recurrent |
| 23 | IRRI_SAH | Lowland | Swarna-Sub1 | 2014WS | RS_DR | 45 | Alpha lattice (5x9) | 2 | F_8_ | 243 | 1003 | 412 | Foreground, Recurrent |
| 24 | IRRI_SAH | Lowland | Swarna-Sub1 | 2014WS | NS | 75 | Alpha lattice (5x15) | 2 | F_8_ | 7180 | 7194 | 1467 | Foreground, Recurrent |
| 25 | IRRI_SAH | Lowland | Swarna-Sub1 | 2014WS | RS_DR | 75 | Alpha lattice (5x15) | 2 | F_8_ | 213 | 634 | 275 | Foreground, Recurrent |
| 26 | IRRI_SAH | Lowland | Swarna-Sub1 | 2015DS | NS | 45 | Augmented RCBD | - | F_8_ | 3323 | 5020 | 1106 | Foreground, Recurrent |
| 27 | IRRI_SAH | Lowland | Swarna-Sub1 | 2015DS | RS_DR | 45 | RCBD | 2 | F_8_ | 349 | 1510 | 477 | Foreground, Recurrent |
| 28 | IRRI_SAH | Lowland | Swarna-Sub1 | 2015WS | NS | 32 | RCBD | 2 | F_8_ | 6640 | 6096 | 1078 | Foreground, Recurrent |
| 29 | IRRI_SAH | Lowland | Swarna-Sub1 | 2015WS | RS_DR | 32 | RCBD | 2 | F_8_ | 0 | 806 | 335 | Foreground, Recurrent |
| 30 | IRRI_HQ | Lowland | IR64-Sub1 | 2013WS | NS | 467 | Augmented RCBD | - | BC_2_F_3_ | 3620 | 3853 | 1390 | Foreground, Recurrent, Background |
| 31 | IRRI_HQ | Lowland | IR64-Sub1 | 2013WS | RS_DR | 467 | Augmented RCBD | - | BC_2_F_3_ | 2305 | 2998 | 818 | Foreground, Recurrent, Background |
| 32 | IRRI_HQ | Lowland | IR64-Sub1 | 2014DS | NS | 190 | Alpha lattice (5x38) | 2 | BC_2_F_4_ | 6066 | 7181 | 1364 | Foreground, Recurrent, Background |
| 33 | IRRI_HQ | Lowland | IR64-Sub1 | 2014DS | RS_DR | 190 | Alpha lattice (5x38) | 2 | BC_2_F_4_ | 87 | 277 | 150 | Foreground, Recurrent, Background |
| 34 | IRRI_HQ | Lowland | IR64-Sub1 | 2014WS | NS | 40 | Augmented RCBD | - | BC_2_F_5_ | 3139 | 3024 | 790 | Foreground, Recurrent, Background |
| 35 | IRRI_HQ | Lowland | IR64-Sub1 | 2015DS | NS | 40 | Augmented RCBD | - | BC_2_F_6_ | 5099 | 4870 | 890 | Foreground, Recurrent, Background |
| 36 | IRRI_HQ | Lowland | IR64-Sub1 | 2015DS | RS_DR | 40 | Alpha lattice (8x5) | 2 | BC_2_F_6_ | 0 | 862 | 225 | Foreground, Recurrent, Background |
| 37 | IRRI_HQ | Lowland | IR64-Sub1 | 2015WS | NS | 18 | Alpha lattice (2x7) | 2 | BC_2_F_7_ | 3849 | 3943 | 356 | Foreground, Recurrent, Background |
| 38 | IRRI_HQ | Lowland | IR64-Sub1 | 2016DS | NS | 14 | Alpha lattice (2x7) | 2 | BC_2_F_8_ | 3956 | 3794 | 672 | Foreground, Recurrent, Background |
| 39 | IRRI_HQ | Lowland | IR64-Sub1 | 2016DS | RS_DR | 14 | Alpha lattice (2x7) | 2 | BC_2_F_8_ | 34 | 328 | 210 | Foreground, Recurrent, Background |
| 40 | IRRI_HQ | Lowland | IR64-Sub1 | 2017DS | NS | 6 | RCBD | 2 | BC_2_F_8_ | 5910 | 6224 | 802 | Foreground, Recurrent, Background |
| 41 | IRRI_HQ | Lowland | IR64-Sub1 | 2017DS | RS_DR | 6 | RCBD | 2 | BC_2_F_8_ | 1029 | 2305 | 688 | Foreground, Recurrent, Background |
| 42 | IRRI_HQ | Lowland | Samba Mahsuri | 2013DS | NS | 45 | Augmented RCBD | - | BC_1_F_3_ | 2742 | 2395 | 705 | Foreground, Recurrent, Background |
| 43 | IRRI_HQ | Lowland | Samba Mahsuri | 2013DS | RS_DR | 45 | Augmented RCBD | - | BC_1_F_3_ | 0 | 1366 | 334 | Foreground, Recurrent, Background |
| 44 | IRRI_HQ | Lowland | Samba Mahsuri | 2014DS | NS | 644 | Augmented RCBD | - | BC_1_F_5_ | 7594 | 8133 | 1472 | Foreground, Recurrent, Background |
| 45 | IRRI_HQ | Lowland | Samba Mahsuri | 2014DS | RS_DR | 644 | Alpha lattice (4x161) | 2 | BC_1_F_5_ | 141 | 850 | 544 | Foreground, Recurrent, Background |
| 46 | IRRI_HQ | Lowland | Samba Mahsuri | 2014WS | NS | 70 | Alpha lattice (7x10) | 2 | BC_1_F_6_ | 2137 | 3038 | 715 | Foreground, Recurrent, Background |
| 47 | IRRI_HQ | Lowland | Samba Mahsuri | 2015DS | NS | 148 | Augmented RCBD | - | BC_1_F_7_ | 6451 | 6144 | 1208 | Foreground, Recurrent, Background |
| 48 | IRRI_HQ | Lowland | Samba Mahsuri | 2015WS | NS | 70 | Alpha lattice (10x7) | 2 | BC_1_F_8_ | 1945 | 4180 | 569 | Foreground, Recurrent, Background |
| 49 | IRRI_HQ | Lowland | Samba Mahsuri | 2016DS | NS | 18 | Alpha lattice (6x3) | 2 | BC_1_F_8_ | 4051 | 5198 | 798 | Foreground, Recurrent, Background |
| 50 | IRRI_HQ | Lowland | Samba Mahsuri | 2016DS | RS_DR | 18 | Alpha lattice (6x3) | 2 | BC_1_F_8_ | 39 | 227 | 144 | Foreground, Recurrent, Background |
| 51 | IRRI_HQ | Lowland | Samba Mahsuri | 2017DS | NS | 2 | RCBD | 2 | BC_1_F_8_ | 5386 | 5380 | 180 | Foreground, Recurrent, Background |
| 52 | IRRI_HQ | Lowland | Samba Mahsuri | 2017DS | RS_DR | 2 | RCBD | 2 | BC_1_F_8_ | 1126 | 2731 | 800 | Foreground, Recurrent, Background |
| 53 | IRRI_SAH | Lowland | Samba Mahsuri | 2014WS | NS | 70 | Alpha lattice (7x10) | 2 | BC_1_F_6_ | 4523 | 6285 | 1113 | Foreground, Recurrent, Background |
| 54 | IRRI_SAH | Lowland | Samba Mahsuri | 2014WS | RS_DR | 70 | Alpha lattice (7x10) | 2 | BC_1_F_6_ | 0 | 352 | 118 | Foreground, Recurrent, Background |
| 55 | IRRI_SAH | Lowland | Samba Mahsuri | 2015DS | NS | 28 | Alpha lattice (4x7) | 2 | BC_1_F_7_ | 7439 | 5858 | 1761 | Foreground, Recurrent, Background |
| 56 | IRRI_SAH | Lowland | Samba Mahsuri | 2015WS | NS | 25 | RCBD | 2 | BC_1_F_8_ | 4166 | 7570 | 1162 | Foreground, Recurrent, Background |
| 57 | IRRI_SAH | Lowland | Samba Mahsuri | 2015WS | RS_DR | 25 | RCBD | 2 | BC_1_F_8_ | 0 | 658 | 112 | Foreground, Recurrent, Background |
| 58 | IRRI_HQ | Lowland | TDK1-Sub1 | 2013WS | RS_DR | 840 | Augmented RCBD | - | BC_2_F_3_ | 421 | 1165 | 87 | Foreground, Recurrent, Background |
| 59 | IRRI_HQ | Lowland | TDK1-Sub1 | 2014DS | NS | 232 | Augmented RCBD | - | BC_2_F_4_ | 6091 | 5886 | 1367 | Foreground, Recurrent, Background |
| 60 | IRRI_HQ | Lowland | TDK1-Sub1 | 2014DS | RS_DR | 232 | Alpha lattice (4x58) | 2 | BC_2_F_4_ | 24 | 1863 | 458 | Foreground, Recurrent, Background |
| 61 | IRRI_HQ | Lowland | TDK1-Sub1 | 2014WS | NS | 48 | Augmented RCBD | - | BC_2_F_5_ | 2167 | 2715 | 488 | Foreground, Recurrent, Background |
| 62 | IRRI_HQ | Lowland | TDK1-Sub1 | 2015DS | NS | 48 | Augmented RCBD | - | BC_2_F_6_ | 6135 | 6091 | 633 | Foreground, Recurrent, Background |
| 63 | IRRI_HQ | Lowland | TDK1-Sub1 | 2015DS | RS_DR | 48 | Alpha lattice (8x6) | 2 | BC_2_F_6_ | 0 | 409 | 172 | Foreground, Recurrent, Background |
| 64 | IRRI_HQ | Lowland | TDK1-Sub1 | 2015WS | NS | 60 | Alpha lattice (10x6) | 2 | BC_2_F_7_ | 3647 | 4583 | 699 | Foreground, Recurrent, Background |
| 65 | IRRI_HQ | Lowland | TDK1-Sub1 | 2016DS | NS | 60 | Alpha lattice (10x6) | 2 | BC_2_F_8_ | 4674 | 4760 | 326 | Foreground, Recurrent, Background |
| 66 | IRRI_HQ | Lowland | TDK1-Sub1 | 2016DS | RS_DR | 60 | Alpha lattice (10x6) | 2 | BC_2_F_8_ | 0 | 198 | 102 | Foreground, Recurrent, Background |
| 67 | IRRI_HQ | Lowland | TDK1-Sub1 | 2017DS | NS | 6 | RCBD | 2 | BC_2_F_8_ | 5736 | 5860 | 910 | Foreground, Recurrent, Background |
| 68 | IRRI_HQ | Lowland | TDK1-Sub1 | 2017DS | RS_DR | 6 | RCBD | 2 | BC_2_F_8_ | 527 | 1916 | 950 | Foreground, Recurrent, Background |
| 69 | IRRI_HQ | Lowland | MR219 | 2013DS | NS | 168 | Alpha lattice (8x21) | 2 | BC_1_F_3_ | 5917 | 6705 | 879 | Foreground, Recurrent, Background |
| 70 | IRRI_HQ | Lowland | MR219 | 2013DS | RS_DR | 168 | Alpha lattice (8x21) | 2 | BC_1_F_3_ | 13 | 781 | 388 | Foreground, Recurrent, Background |
| 71 | IRRI_HQ | Lowland | MR219 | 2013WS | NS | 295 | Augmented RCBD | - | BC_1_F_4_ | 4850 | 4949 | 225 | Foreground, Recurrent, Background |
| 72 | IRRI_HQ | Lowland | MR219 | 2014DS | NS | 616 | Alpha lattice (4x154) | 2 | BC_1_F_5_ | 6519 | 7173 | 512 | Foreground, Recurrent, Background |
| 73 | IRRI_HQ | Lowland | MR219 | 2014DS | RS_DR | 616 | Alpha lattice (4x154) | 2 | BC_1_F_5_ | 0 | 505 | 223 | Foreground, Recurrent, Background |
| 74 | IRRI_HQ | Lowland | MR219 | 2014WS | NS | 70 | Augmented RCBD | - | BC_1_F_6_ | 4205 | 4585 | 298 | Foreground, Recurrent, Background |
| 75 | IRRI_HQ | Lowland | MR219 | 2015DS | NS | 70 | Augmented RCBD | - | BC_1_F_7_ | 6148 | 6663 | 453 | Foreground, Recurrent, Background |
| 76 | IRRI_HQ | Lowland | MR219 | 2015DS | RS_DR | 70 | Alpha lattice (7x10) | 2 | BC_1_F_7_ | 0 | 486 | 146 | Foreground, Recurrent, Background |
| 77 | IRRI_HQ | Lowland | MR219 | 2015WS | NS | 21 | Alpha lattice (3x7) | 2 | BC_1_F_8_ | 4167 | 5413 | 935 | Foreground, Recurrent, Background |
| 78 | IRRI_HQ | Lowland | MR219 | 2016DS | NS | 6 | RCBD | 2 | BC_1_F_8_ | 6518 | 5643 | 1292 | Foreground, Recurrent, Background |
| 79 | IRRI_HQ | Lowland | MR219 | 2016DS | RS_DR | 6 | RCBD | 2 | BC_1_F_8_ | 0 | 903 | 732 | Foreground, Recurrent, Background |
| 80 | IRRI_HQ | Lowland | MR219 | 2017DS | NS | 5 | RCBD | 2 | BC_1_F_8_ | 5768 | 6064 | 808 | Foreground, Recurrent, Background |
| 81 | IRRI_HQ | Lowland | MR219 | 2017DS | RS_DR | 5 | RCBD | 2 | BC_1_F_8_ | 573 | 2675 | 523 | Foreground, Recurrent, Background |
| 82 | IRRI_SAH | Lowland | MR219 | 2014WS | NS | 63 | Alpha lattice (7x9) | 2 | BC_1_F_6_ | 5435 | 6012 | 1015 | Foreground, Recurrent, Background |
| 83 | IRRI_SAH | Lowland | MR219 | 2014WS | RS_DR | 63 | Alpha lattice (7x9) | 2 | BC_1_F_6_ | 203 | 2683 | 1003 | Foreground, Recurrent, Background |
| 84 | IRRI_SAH | Lowland | MR219 | 2015DS | NS | 20 | RCBD | 2 | BC_1_F_7_ | 3969 | 5581 | 2147 | Foreground, Recurrent, Background |
| 85 | IRRI_SAH | Lowland | MR219 | 2015WS | NS | 15 | RCBD | 2 | BC_1_F_8_ | 6338 | 8294 | 1521 | Foreground, Recurrent, Background |
| 86 | IRRI_SAH | Lowland | MR219 | 2016DS | RS_DR | 15 | RCBD | 2 | BC_1_F_8_ | 0 | 1408 | 332 | Foreground, Recurrent, Background |
| 87 | IRRI_HQ | Lowland | Savitri | 2013DS | NS | 1250 | Augmented RCBD | - | F_3_ | 2979 | 4906 | 1123 | Foreground, Recurrent |
| 88 | IRRI_HQ | Lowland | Savitri | 2013WS | NS | 40 | Augmented RCBD | - | F_4_ | 2978 | 3428 | 445 | Foreground, Recurrent |
| 89 | IRRI_HQ | Lowland | Savitri | 2014DS | NS | 100 | Alpha lattice (5x20) | 2 | F_5_ | 7027 | 5932 | 389 | Foreground, Recurrent |
| 90 | IRRI_HQ | Lowland | Savitri | 2014DS | RS_DR | 100 | Alpha lattice (5x20) | 2 | F_5_ | 155 | 800 | 62 | Foreground, Recurrent |
| 91 | IRRI_HQ | Lowland | Savitri | 2014WS | NS | 120 | Augmented RCBD | - | F_6_ | 3301 | 3517 | 77 | Foreground, Recurrent |
| 92 | IRRI_HQ | Lowland | Savitri | 2015DS | NS | 130 | Augmented RCBD | - | F_7_ | 6393 | 5732 | 107 | Foreground, Recurrent |
| 93 | IRRI_HQ | Lowland | Savitri | 2015DS | RS_DR | 130 | Alpha lattice (10x13) | 2 | F_7_ | 48 | 563 | 440 | Foreground, Recurrent |
| 94 | IRRI_HQ | Lowland | Savitri | 2015WS | NS | 100 | Augmented RCBD | - | F_8_ | 5210 | 5312 | 172 | Foreground, Recurrent |
| 95 | IRRI_HQ | Lowland | Savitri | 2016DS | NS | 45 | Alpha lattice (5x9) | 2 | F_8_ | 4749 | 5029 | 442 | Foreground, Recurrent |
| 96 | IRRI_HQ | Lowland | Savitri | 2016DS | RS_DR | 45 | Alpha lattice (5x9) | 2 | F_8_ | 23 | 293 | 257 | Foreground, Recurrent |
| 97 | IRRI_HQ | Lowland | Savitri | 2017DS | NS | 7 | RCBD | 2 | F_8_ | 6574 | 6125 | 1098 | Foreground, Recurrent |
| 98 | IRRI_HQ | Lowland | Savitri | 2017DS | RS_DR | 7 | RCBD | 2 | F_8_ | 421 | 718 | 296 | Foreground, Recurrent |
| 99 | IRRI_HQ | Lowland | MTU1010 | 2012DS | NS | 800 | Alpha lattice (20x40) | 2 | BC_2_F_5_ | 5704 | 5254 | 633 | Foreground, Recurrent, Background |
| 100 | IRRI_HQ | Lowland | MTU1010 | 2012DS | RS_DR | 800 | Alpha lattice (20x40) | 2 | BC_2_F_5_ | 2677 | 2429 | 339 | Foreground, Recurrent, Background |
| 101 | IRRI_SAH | Lowland | MTU1010 | 2012WS | RS_DR | 300 | Alpha lattice (15x20) | 2 | BC_2_F_6_ | 3367 | 2970 | 1274 | Foreground, Recurrent, Background |
| 102 | IRRI_SAH | Lowland | MTU1010 | 2013WS | NS | 50 | Augmented RCBD | - | BC_2_F_7_ | 7975 | 6477 | 2710 | Foreground, Recurrent, Background |
| 103 | IRRI_SAH | Lowland | MTU1010 | 2014DS | NS | 49 | Alpha lattice (7x7) | 2 | BC_2_F_8_ | 5821 | 5541 | 1330 | Foreground, Recurrent, Background |
| 104 | IRRI_SAH | Lowland | MTU1010 | 2014DS | RS_DR | 49 | Alpha lattice (7x7) | 2 | BC_2_F_8_ | 208 | 244 | 333 | Foreground, Recurrent, Background |
| 105 | IRRI_SAH | Lowland | MTU1010 | 2014WS | NS | 16 | Alpha lattice (4x4) | 2 | BC_2_F_8_ | 4929 | 4661 | 1446 | Foreground, Recurrent, Background |
| 106 | IRRI_SAH | Lowland | MTU1010 | 2014WS | RS_DR | 16 | Alpha lattice (4x4) | 2 | BC_2_F_8_ | 498 | 960 | 569 | Foreground, Recurrent, Background |
| 107 | IRRI_SAH | Lowland | MTU1010 | 2015WS | NS | 6 | RCBD | 2 | BC_2_F_8_ | 8922 | 9698 | 1196 | Foreground, Recurrent, Background |
| 108 | IRRI_SAH | Lowland | MTU1010 | 2015WS | RS_DR | 6 | RCBD | 2 | BC_2_F_8_ | 1377 | 1451 | 65 | Foreground, Recurrent, Background |
| 109 | IRRI_HQ | Lowland | Swarna | 2013WS | NS | 80 | Alpha lattice (10x8) | 2 | BC_3_F_7_ | 6857 | 6954 | 1627 | Foreground, Recurrent, Background |
| 110 | IRRI_HQ | Lowland | Swarna | 2013WS | RS_DR | 80 | Alpha lattice (10x8) | 2 | BC_3_F_7_ | 101 | 1285 | 864 | Foreground, Recurrent, Background |
| 111 | IRRI_HQ | Lowland | Swarna | 2014DS | NS | 30 | Alpha lattice (10x68) | 3 | BC_3_F_8_ | 7136 | 5344 | 1568 | Foreground, Recurrent, Background |
| 112 | IRRI_HQ | Lowland | Swarna | 2014DS | RS_DR | 30 | Alpha lattice (10x68) | 3 | BC_3_F_8_ | 0 | 341 | 244 | Foreground, Recurrent, Background |
| 113 | IRRI_SAH | Lowland | Swarna | 2012WS | RS_DR | 420 | Alpha lattice (14x30) | 2 | BC_3_F_5_ | 441 | 1166 | 462 | Foreground, Recurrent, Background |
| 114 | IRRI_SAH | Lowland | Swarna | 2013DS | NS | 144 | Augmented RCBD | - | BC_3_F_6_ | 5108 | 7111 | 3633 | Foreground, Recurrent, Background |
| 115 | IRRI_SAH | Lowland | Swarna | 2013WS | NS | 104 | Alpha lattice (8x13) | 2 | BC_3_F_7_ | 5623 | 7635 | 2332 | Foreground, Recurrent, Background |
| 116 | IRRI_SAH | Lowland | Swarna | 2013WS | NS | 50 | Augmented RCBD | - | BC_3_F_7_ | 6875 | 8918 | 435 | Foreground, Recurrent, Background |
| 117 | IRRI_SAH | Lowland | Swarna | 2014DS | NS | 16 | Alpha lattice (4x4) | 2 | BC_3_F_8_ | 4777 | 4723 | 1938 | Foreground, Recurrent, Background |
| 118 | IRRI_SAH | Lowland | Swarna | 2014DS | RS_DR | 16 | Alpha lattice (4x4) | 2 | BC_3_F_8_ | 41 | 1646 | 680 | Foreground, Recurrent, Background |
| 119 | IRRI_SAH | Lowland | Swarna | 2014DS | NS | 110 | Alpha lattice (10x11) | 2 | BC_3_F_8_ | 4667 | 4359 | 1651 | Foreground, Recurrent, Background |
| 120 | IRRI_SAH | Lowland | Swarna | 2014DS | RS_DR | 110 | Alpha lattice (10x11) | 2 | BC_3_F_8_ | 267 | 1129 | 783 | Foreground, Recurrent, Background |
| 121 | IRRI_SAH | Lowland | Swarna | 2014WS | NS | 16 | Alpha lattice (4x4) | 2 | BC_3_F_8_ | 6421 | 5883 | 1894 | Foreground, Recurrent, Background |
| 122 | IRRI_SAH | Lowland | Swarna | 2014WS | RS_DR | 16 | Alpha lattice (4x4) | 2 | BC_3_F_8_ | 551 | 1239 | 412 | Foreground, Recurrent, Background |
| 123 | IRRI_SAH | Lowland | Swarna | 2015DS | NS | 8 | RCBD | 2 | BC_3_F_8_ | 5544 | 5692 | 1203 | Foreground, Recurrent, Background |
| 124 | IRRI_SAH | Lowland | Swarna | 2015WS | NS | 7 | RCBD | 2 | BC_3_F_8_ | 6672 | 8066 | 1108 | Foreground, Recurrent, Background |
| 125 | IRRI_SAH | Lowland | Swarna | 2015WS | RS_DR | 7 | RCBD | 2 | BC_3_F_8_ | 0 | 1648 | 257 | Foreground, Recurrent, Background |
| 126 | IRRI_SAH | Lowland | MTU1010 | 2016DS | RS_HT | 45 | Alpha lattice (5x9) | 2 | F_5_ | 1877 | 3585 | 507 | Foreground, Recurrent |
| 127 | IRRI_SAH | Lowland | MTU1010 | 2017DS | NS | 45 | Alpha lattice (5x9) | 2 | F_6_ | 4988 | 5026 | 677 | Foreground, Recurrent |
| 128 | IRRI_SAH | Lowland | MTU1010 | 2017DS | RS_DR | 45 | Alpha lattice (5x9) | 2 | F_6_ | 495 | 1985 | 405 | Foreground, Recurrent |
| 129 | IRRI_SAH | Lowland | MTU1010 | 2017DS | RS_HT | 45 | Alpha lattice (5x9) | 2 | F_6_ | 2353 | 3611 | 266 | Foreground, Recurrent |
| 130 | IRRI_SAH | Lowland | MTU1010 | 2017WS | NS | 45 | Alpha lattice (5x9) | 2 | F_7_ | 5137 | 5176 | 697 | Foreground, Recurrent |
| 131 | IRRI_SAH | Lowland | MTU1010 | 2017WS | RS_DR | 45 | Alpha lattice (5x9) | 2 | F_7_ | 621 | 1927 | 211 | Foreground, Recurrent |
| 132 | IRRI_SAH | Lowland | Sahbhagidhan | 2017DS | NS | 3 | RCBD | 2 | BC_3_F_4_ | 4332 | 5318 | 536 | Foreground, Recurrent, Background |
| 133 | IRRI_SAH | Lowland | Sahbhagidhan | 2017WS | NS | 3 | RCBD | 2 | BC_3_F_5_ | 4622 | 5863 | 541 | Foreground, Recurrent, Background |
| 134 | IRRI_HQ | Upland | Vandana | 2010DS | NS | 500 | Augmented RCBD | - | BC_3_F_3_ | 4061 | 3787 | 924 | Foreground, Recurrent, Background |
| 135 | IRRI_HQ | Upland | Vandana | 2010WS | NS | 150 | RCBD | 2 | BC_3_F_4_ | 3636 | 3631 | 547 | Foreground, Recurrent, Background |
| 136 | IRRI_HQ | Upland | Vandana | 2010WS | RS_DR | 150 | RCBD | 2 | BC_3_F_4_ | 2404 | 2408 | 334 | Foreground, Recurrent, Background |
| 137 | IRRI_HQ | Upland | Vandana | 2011DS | NS | 40 | Alpha lattice (4x10) | 2 | BC_3_F_5_ | 2127 | 2460 | 406 | Foreground, Recurrent, Background |
| 138 | IRRI_HQ | Upland | Vandana | 2011DS | RS_DR | 40 | Alpha lattice (4x10) | 2 | BC_3_F_5_ | 27 | 286 | 59 | Foreground, Recurrent, Background |
| 139 | IRRI_HQ | Upland | Vandana | 2012DS | NS | 10 | RCBD | 2 | BC_3_F_6_ | 3992 | 4312 | 531 | Foreground, Recurrent, Background |
| 140 | IRRI_HQ | Upland | Vandana | 2012DS | RS_DR | 10 | RCBD | 2 | BC_3_F_6_ | 1393 | 1754 | 191 | Foreground, Recurrent, Background |
| 141 | IRRI_HQ | Upland | Vandana | 2013DS | NS | 4 | RCBD | 2 | BC_3_F_7_ | 4307 | 2118 | 222 | Foreground, Recurrent, Background |
| 142 | IRRI_HQ | Upland | Vandana | 2013DS | RS_DR | 4 | RCBD | 2 | BC_3_F_7_ | 225 | 345 | 44 | Foreground, Recurrent, Background |
| 143 | IRRI_SAH | Upland | Vandana | 2013WS | NS | 6 | RCBD | 3 | BC_3_F_8_ | 6058 | 6072 | 651 | Foreground, Recurrent, Background |
| 144 | IRRI_SAH | Upland | Vandana | 2013WS | RS_DR | 6 | RCBD | 2 | BC_3_F_8_ | 1805 | 1713 | 394 | Foreground, Recurrent, Background |
| 145 | IRRI_SAH | Upland | Vandana | 2014WS | NS | 5 | RCBD | 2 | BC_3_F_8_ | 4855 | 4575 | 388 | Foreground, Recurrent, Background |
| 146 | IRRI_SAH | Upland | Vandana | 2015DS | RS_DR | 5 | RCBD | 3 | BC_3_F_8_ | 1095 | 1165 | 197 | Foreground, Recurrent, Background |
| 147 | IRRI_SAH | Upland | Vandana | 2015WS | RS_DR | 2 | RCBD | 2 | BC_3_F_8_ | 1945 | 2345 | 221 | Foreground, Recurrent, Background |
| 148 | IRRI_HQ | Upland | Anjali | 2013DS | NS | 47 | RCBD | 2 | BC_3_F_4_ | 5543 | 5121 | 556 | Foreground, Recurrent, Background |
| 149 | IRRI_HQ | Upland | Anjali | 2013DS | RS_DR | 47 | RCBD | 2 | BC_3_F_4_ | 0 | 83 | 44 | Foreground, Recurrent, Background |
| 150 | IRRI_HQ | Upland | Anjali | 2014DS | NS | 350 | Alpha lattice (10x35) | 2 | BC_3_F_6_ | 3237 | 3197 | 717 | Foreground, Recurrent, Background |
| 151 | IRRI_HQ | Upland | Anjali | 2014DS | RS_DR | 350 | Alpha lattice (10x35) | 2 | BC_3_F_6_ | 97 | 498 | 252 | Foreground, Recurrent, Background |
| 152 | IRRI_HQ | Upland | Anjali | 2014WS | RS_DR | 30 | RCBD | 2 | BC_3_F_6_ | 1922 | 1736 | 359 | Foreground, Recurrent, Background |
| 153 | IRRI_HQ | Upland | Anjali | 2015DS | NS | 35 | Alpha lattice (7x5) | 2 | BC_3_F_7_ | 5118 | 4194 | 989 | Foreground, Recurrent, Background |
| 154 | IRRI_HQ | Upland | Anjali | 2015DS | RS_DR | 35 | Alpha lattice (7x5) | 2 | BC_3_F_7_ | 483 | 968 | 321 | Foreground, Recurrent, Background |
| 155 | IRRI_HQ | Upland | Anjali | 2016DS | NS | 5 | RCBD | 2 | BC_3_F_8_ | 3970 | 3358 | 226 | Foreground, Recurrent, Background |
| 156 | IRRI_HQ | Upland | Anjali | 2016DS | RS_DR | 5 | RCBD | 2 | BC_3_F_8_ | 491 | 763 | 191 | Foreground, Recurrent, Background |
| 157 | IRRI_HQ | Upland | Anjali | 2016WS | NS | 5 | RCBD | 2 | BC_3_F_8_ | 3666 | 3751 | 122 | Foreground, Recurrent, Background |
| 158 | IRRI_HQ | Upland | Anjali | 2016WS | RS_DR | 5 | RCBD | 2 | BC_3_F_8_ | 1271 | 1451 | 56 | Foreground, Recurrent, Background |
| 159 | IRRI_SAH | Upland | Anjali | 2013WS | RS_DR | 560 | Augmented RCBD | - | BC_3_F_5_ | 270 | 2141 | 637 | Foreground, Recurrent, Background |
| 160 | IRRI_SAH | Upland | Anjali | 2014WS | NS | 30 | Alpha lattice (5x6) | 2 | BC_3_F_6_ | 3265 | 3347 | 948 | Foreground, Recurrent, Background |
| 161 | IRRI_SAH | Upland | Anjali | 2015DS | NS | 15 | RCBD | 2 | BC_3_F_7_ | 3340 | 2925 | 379 | Foreground, Recurrent, Background |
| 162 | IRRI_SAH | Upland | Anjali | 2015WS | RS_DR | 5 | RCBD | 2 | BC_3_F_8_ | 1677 | 1463 | 333 | Foreground, Recurrent, Background |
| 163 | IRRI_HQ | Upland | Kalinga III | 2013DS | NS | 680 | Alpha lattice (10x68) | 2 | F_3_ | 2050 | 2150 | 445 | Foreground, Recurrent |
| 164 | IRRI_HQ | Upland | Kalinga III | 2013DS | RS_DR | 680 | Alpha lattice (10x68) | 2 | F_3_ | 30 | 221 | 127 | Foreground, Recurrent |
| 165 | IRRI_HQ | Upland | Kalinga III | 2014DS | NS | 650 | Augmented RCBD | - | F_4_ | 2795 | 2150 | 90 | Foreground, Recurrent |
| 166 | IRRI_HQ | Upland | Kalinga III | 2014DS | RS_DR | 650 | Augmented RCBD | - | F_4_ | 22 | 352 | 44 | Foreground, Recurrent |
| 167 | IRRI_HQ | Upland | Kalinga III | 2014WS | NS | 40 | Augmented RCBD | - | F_5_ | 4692 | 2058 | 179 | Foreground, Recurrent |
| 168 | IRRI_HQ | Upland | Kalinga III | 2015DS | NS | 15 | RCBD | 2 | F_6_ | 5697 | 3873 | 1087 | Foreground, Recurrent |
| 169 | IRRI_HQ | Upland | Kalinga III | 2015DS | RS_DR | 15 | RCBD | 2 | F_6_ | 195 | 615 | 190 | Foreground, Recurrent |
| 170 | IRRI_HQ | Upland | Kalinga III | 2015WS | NS | 15 | RCBD | 2 | F_7_ | 3746 | 3936 | 437 | Foreground, Recurrent |
| 171 | IRRI_HQ | Upland | Kalinga III | 2015WS | RS_DR | 15 | RCBD | 2 | F_7_ | 0 | 407 | 177 | Foreground, Recurrent |
| 172 | IRRI_HQ | Upland | Kalinga III | 2016DS | NS | 15 | RCBD | 2 | F_7_ | 3746 | 3885 | 608 | Foreground, Recurrent |
| 173 | IRRI_HQ | Upland | Kalinga III | 2016DS | RS_DR | 15 | RCBD | 2 | F_7_ | 0 | 390 | 85 | Foreground, Recurrent |
| 174 | IRRI_HQ | Upland | Kalinga III | 2016WS | NS | 15 | RCBD | 2 | F_7_ | 4426 | 3942 | 831 | Foreground, Recurrent |
| 175 | IRRI_HQ | Upland | Kalinga III | 2016WS | RS_DR | 15 | RCBD | 2 | F_7_ | 0 | 1007 | 370 | Foreground, Recurrent |
| 176 | IRRI_SAH | Upland | Kalinga III | 2014WS | NS | 40 | Augmented RCBD | - | F_5_ | 5819 | 4296 | 254 | Foreground, Recurrent |
| 177 | IRRI_SAH | Upland | Kalinga III | 2015DS | NS | 50 | Augmented RCBD | - | F_6_ | 4189 | 3637 | 101 | Foreground, Recurrent |
| 178 | IRRI_SAH | Upland | Kalinga III | 2015DS | RS_DR | 140 | Augmented RCBD | - | F_6_ | 4487 | 3253 | 91 | Foreground, Recurrent |
| 179 | IRRI_SAH | Upland | Kalinga III | 2015WS | NS | 62 | Augmented RCBD | - | F_7_ | 3900 | 4166 | 78 | Foreground, Recurrent |
| 180 | IRRI_SAH | Upland | Kalinga III | 2015WS | RS_DR | 106 | Augmented RCBD | - | F_7_ | 2538 | 2158 | 71 | Foreground, Recurrent |
| 181 | IRRI_SAH | Upland | Kalinga III | 2016DS | NS | 10 | Augmented RCBD | - | F_7_ | 2300 | 4790 | 635 | Foreground, Recurrent |

**Table S2.** The detailed information on the markers used to detect the introgressed region in different genetic backgrounds.

| Background | QTLs with marker interval | References |
| --- | --- | --- |
| Swarna-Sub1 | *qDTY_1.1_* (RM315, RM11943, RM12023, RM12091, RM12233) | Sandhu et al. 2019 |
|  | *qDTY_2.1_* (RM5791, RM521, RM3549, RM324, RM6374, RM424) |  |
|  | *qDTY_3.1_* (RM15791, RM416, RM16030, RM520) |  |
|  | *Sub1* (ART5, SC3) |  |
| IR 64-Sub1 | *qDTY_1.1_* (RM11943, RM12023, RM12233) | Not published |
|  | *qDTY_1.2_* (RM212, RM3825, RM315) |  |
|  | *qDTY_2.2_* (RM154, RM236, RM279, RM555, OSR17, RM492) |  |
|  | *qDTY_2.3_* (RM3212, RM573, RM1367) |  |
|  | *qDTY_3.2_* (RM523, RM22, RM545) |  |
|  | *qDTY_4.1_* (RM518, RM335, RM16368) |  |
|  | *qDTY_12.1_* (RM28048, RM28099, INDEL8, RM28130) |  |
|  | *Sub1* (ART5, SC3) |  |
| TDK1-Sub1 | *qDTY_3.1_*: RM55, RM168, RM186, RM293, RM468 | Dixit et al. 2017 |
|  | *qDTY_6.1_*:RM204, RM217, RM508, RM586, RM587 |  |
|  | *qDTY_6.2_*: RM3, RM541 |  |
|  | *Sub1* (ART5, SC3) |  |
| Savitri | *qDTY_3.2_*: RM231, RM517 | Yadaw et al. 2013 |
|  | *qDTY_12.1_*: RM28048, RM511, RM28199, RM28166 |  |
| Sambha Mahsuri | *qDTY_2.2_*: RM236, RM279, RM555 | Sandhu et al. 2018 |
|  | *qDTY_4.1_*: RM518, RM335, RM16368 |  |
| MR219 | *qDTY_2.2_*: RM236, RM279, RM12460 | Shamsudin et al. 2016 |
|  | *qDTY_3.1_*: RM416, RM16030, RM520 |  |
|  | *qDTY_12.1_*: RM28048, RM511, RM28099, RM28166, CG29430, indel8, RM28130 |  |
| Swarna | *qDTY_3.2_* (RM523, RM22, RM545) | Not published |
| MTU1010 | *qDTY_3.1_*: RM55, RM168, RM186, RM293, RM468 | Not published |
|  | *qDTY_6.1_*:RM204, RM217, RM508, RM586, RM587 |  |
|  | *qDTY_6.2_*: RM3, RM541 |  |
|  | *qDTY_2.2_*: RM236, RM279, RM555 |  |
|  | *qDTY_4.1_*: RM518, RM335, RM16368 |  |
| Anjali | *qDTY_3.1_*: RM416, RM16030, RM520 | Not published |
|  | *qDTY_12.1_*: RM28048, RM28130, RM28099, CG29430, indel8 |  |
| Kalinga III | *qDTY_12.1_*: RM28048, RM28130, RM28099, CG29430, indel8 | Not published |
| Vandana | *qDTY_12.1_*: RM28048, RM28130, RM28099, CG29430, indel8 | Bernier et al. 2007 |
| Sahbhagi dhan | nksbadh2 | Singh et al. 2011 |


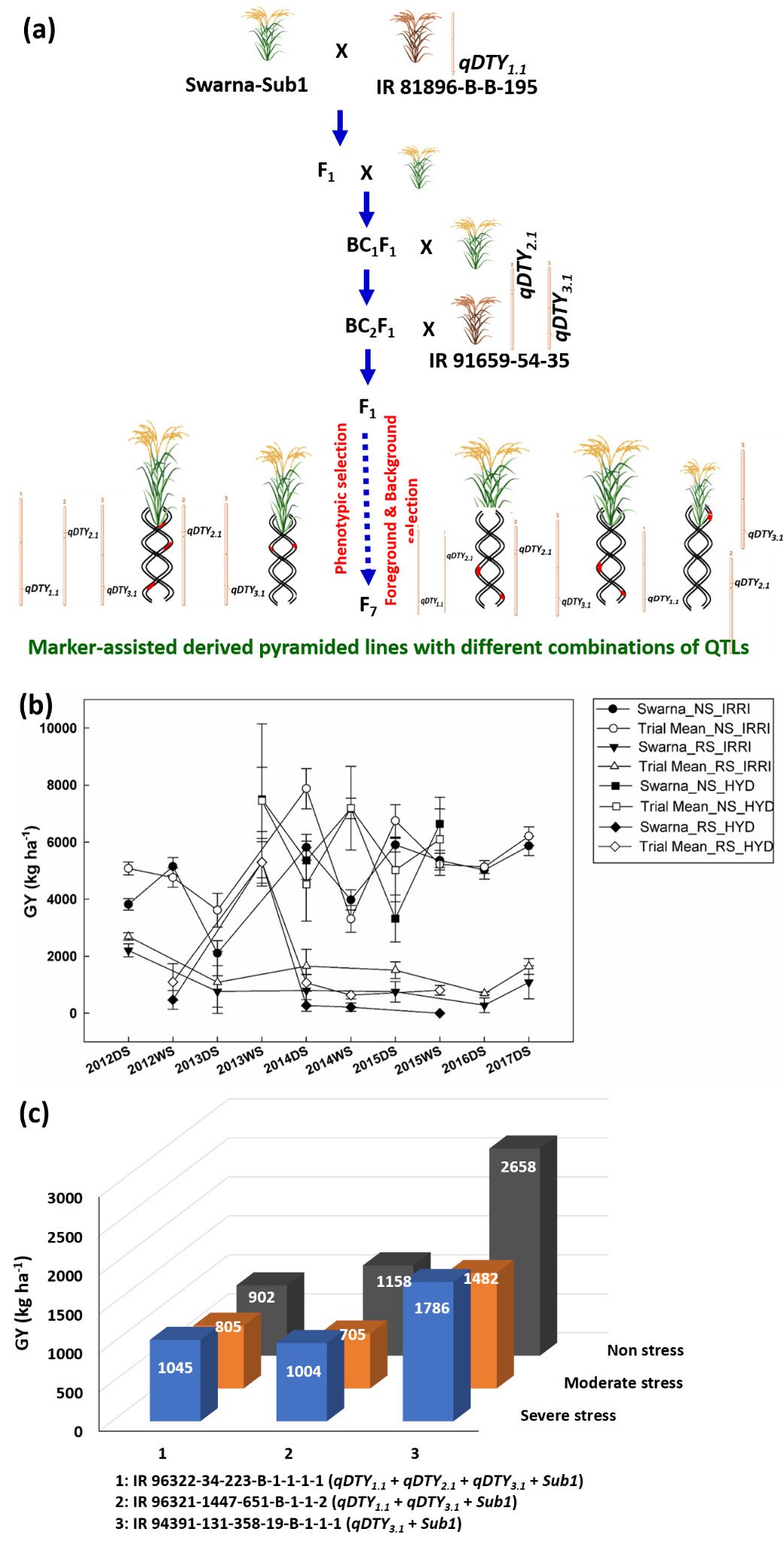


**Figure S1.** **A:** Marker-assisted breeding scheme for the development of pyramided lines in Swarna-Sub1 background. **B:** The mean grain yield performance of introgression lines in comparison with Swarna under NS and RS across seasons at IRRI, Philippines and SAH (Hyderabad, India). **C:** mean grain yield advantage of pyramided lines over Swarna under non-stress, moderate stress and severe stress.


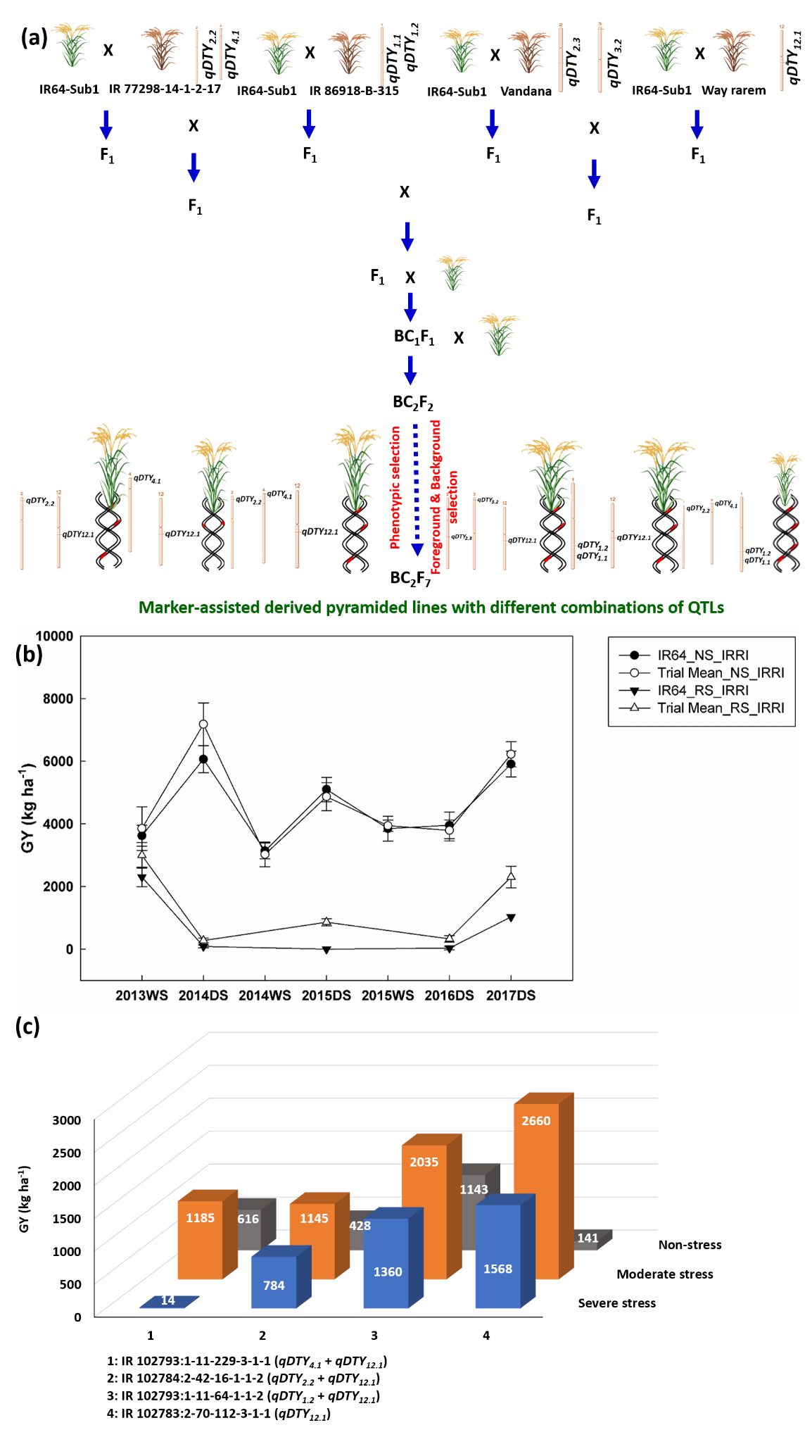


**Figure S2.** **A:** Marker-assisted breeding scheme for the development of pyramided lines in IR64-Sub1 background. **B:** The mean grain yield performance of introgression lines in comparison with IR64 under NS and RS across seasons at IRRI, Philippines. **C:** mean grain yield advantage of pyramided lines over IR64 under non-stress, moderate stress and severe stress.


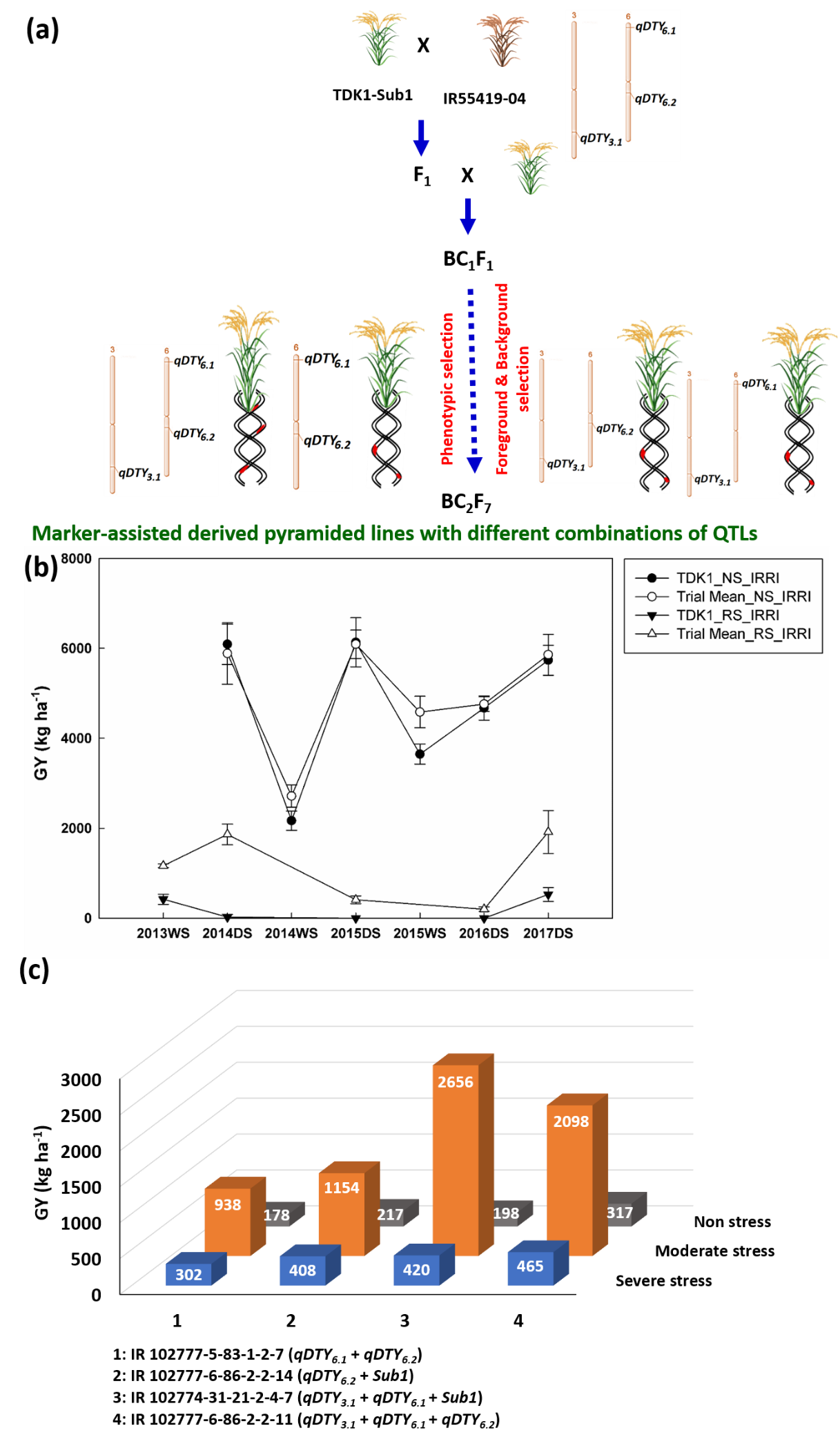


**Figure S3.** **A:** Marker-assisted breeding scheme for the development of pyramided lines in TDK1-Sub1 background. **B:** The mean grain yield performance of introgression lines in comparison with TDK1 under NS and RS across seasons at IRRI, Philippines. **C:** mean grain yield advantage of pyramided lines over TDK1 under non-stress, moderate stress and severe stress.


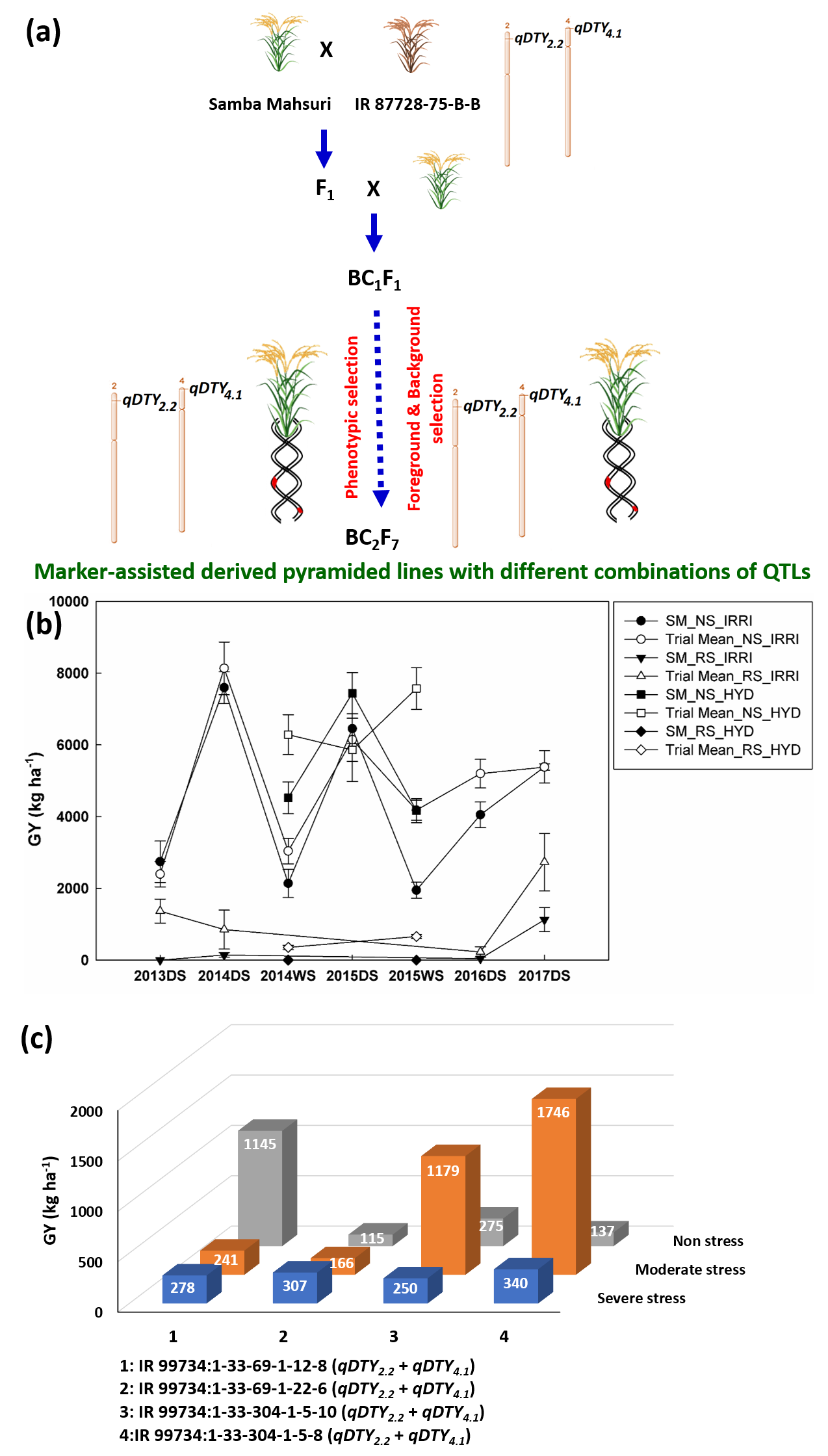


**Figure S4.** **A:** Marker-assisted breeding scheme for the development of pyramided lines in Samba Mahsuri background. **B:** The mean grain yield performance of introgression lines in comparison with Samba Mahsuri under NS and RS across seasons at IRRI, Philippines and SAH (Hyderabad, India). **C:** mean grain yield advantage of pyramided lines over Samba Mahsuri under non-stress, moderate stress and severe stress.


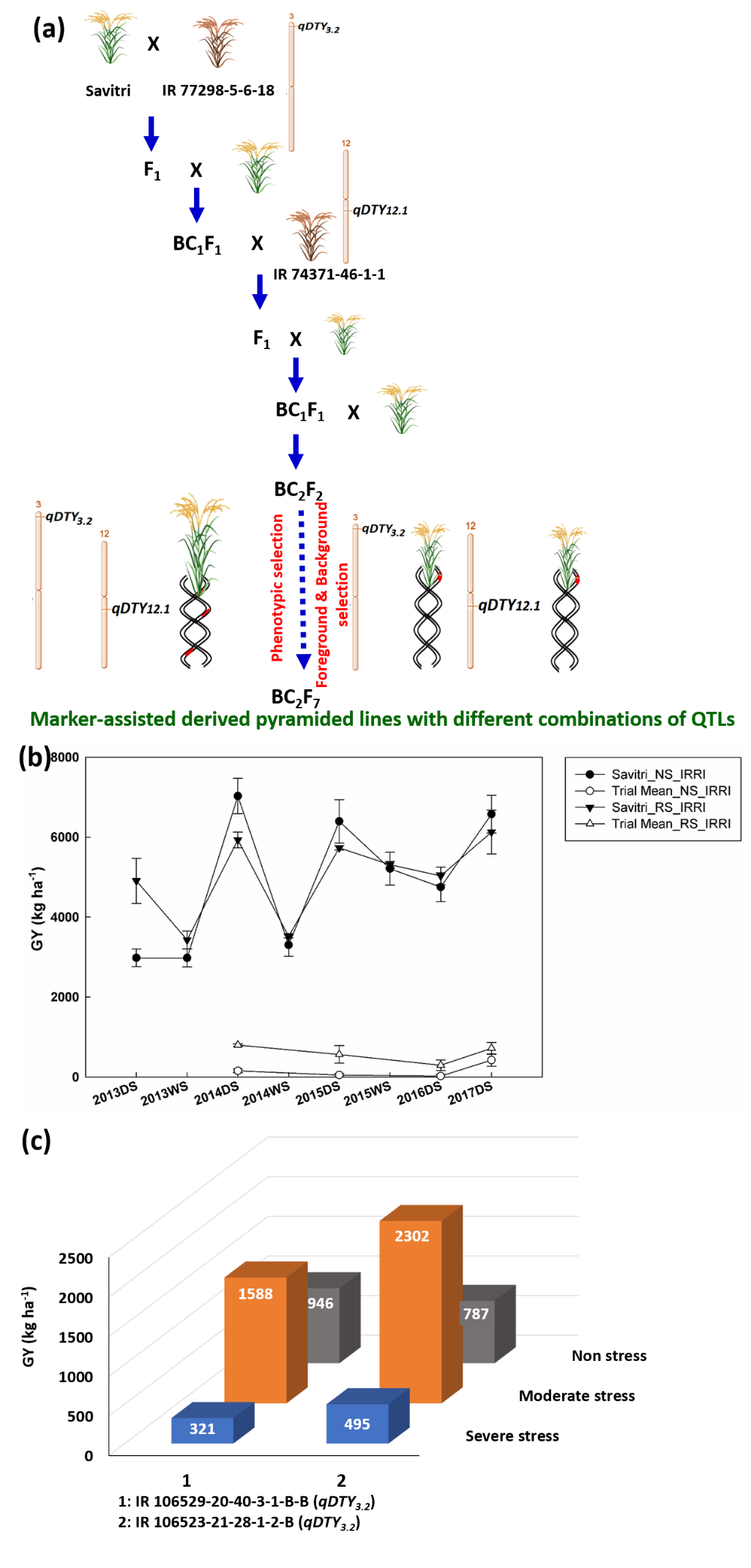


**Figure S5.** **A:** Marker-assisted breeding scheme for the development of pyramided lines in Savitri background. **B:** The mean grain yield performance of introgression lines in comparison with Savitri under NS and RS across seasons at IRRI, Philippines. **C:** mean grain yield advantage of pyramided lines over Savitri under non-stress, moderate stress and severe stress.


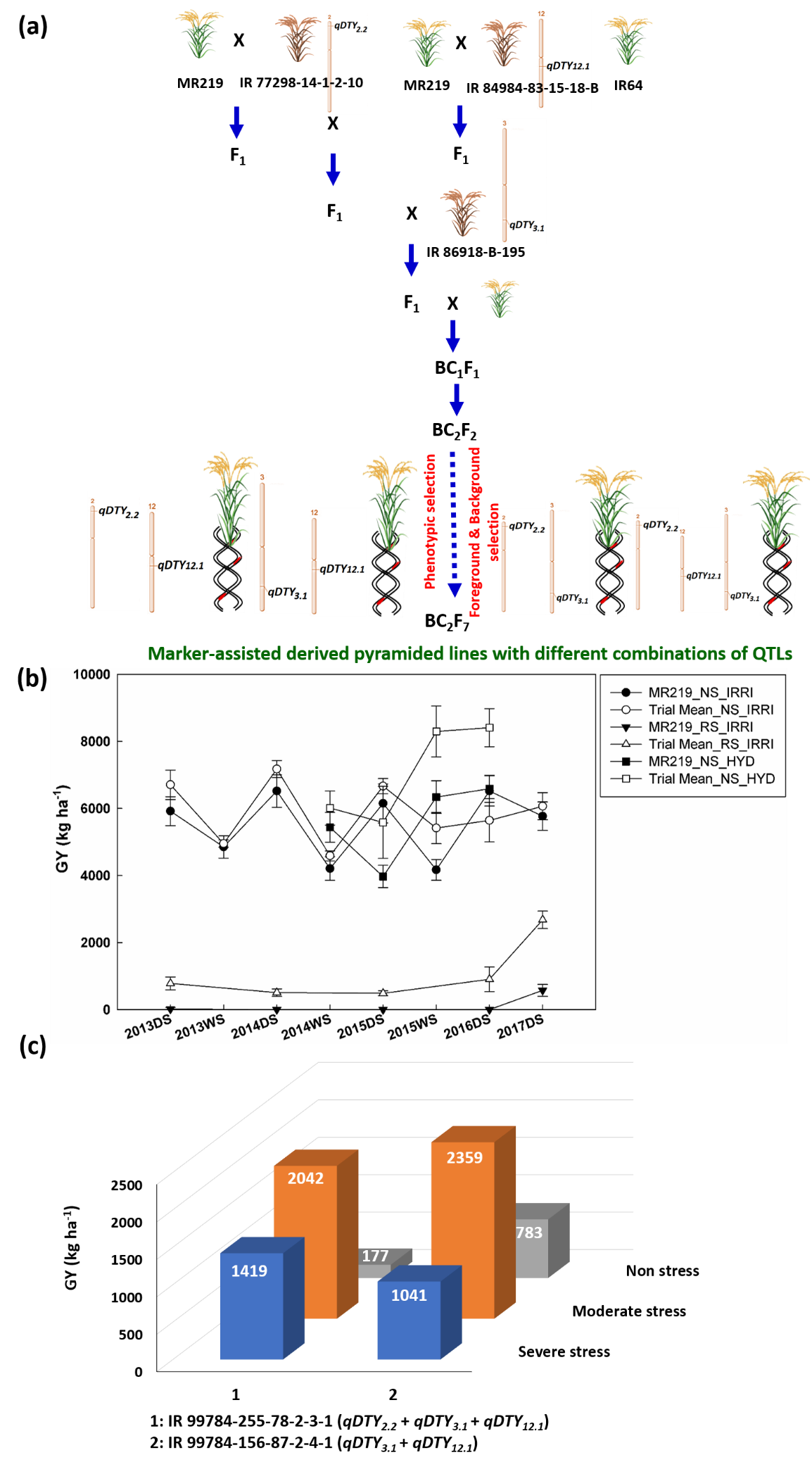


**Figure S6.** **A:** Marker-assisted breeding scheme for the development of pyramided lines in MR219 background. **B:** The mean grain yield performance of introgression lines in comparison with MR219 under NS and RS across seasons at IRRI, Philippines and SAH (Hyderabad, India). **C:** mean grain yield advantage of pyramided lines over MR219 under non-stress, moderate stress and severe stress.


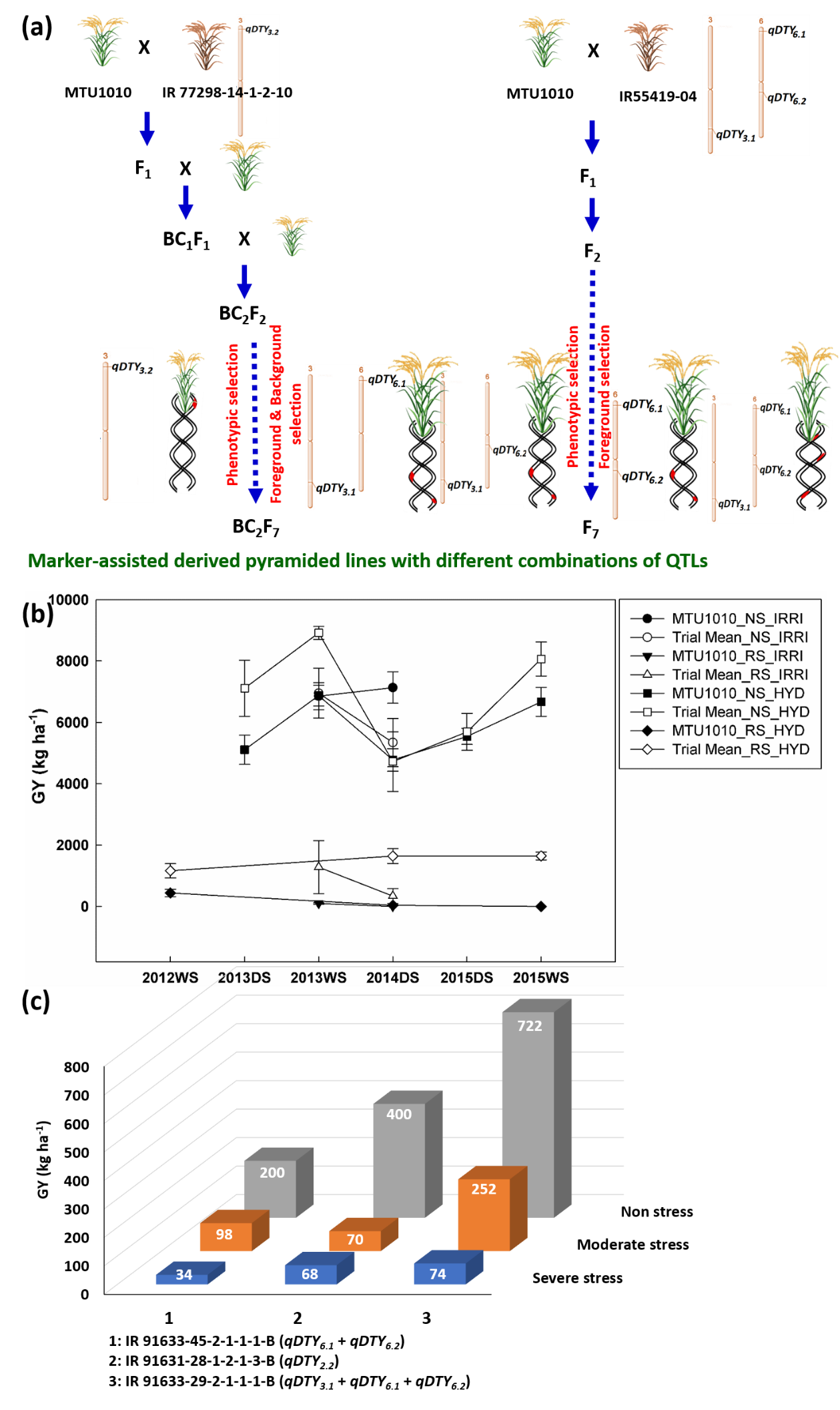


**Figure S7.** **A:** Marker-assisted breeding scheme for the development of pyramided lines in MTU1010 background. **B:** The mean grain yield performance of introgression lines in comparison with MTU1010 under NS and RS across seasons at IRRI, Philippines and SAH (Hyderabad, India). **C:** mean grain yield advantage of pyramided lines over MTU1010 under non-stress, moderate stress and severe stress.


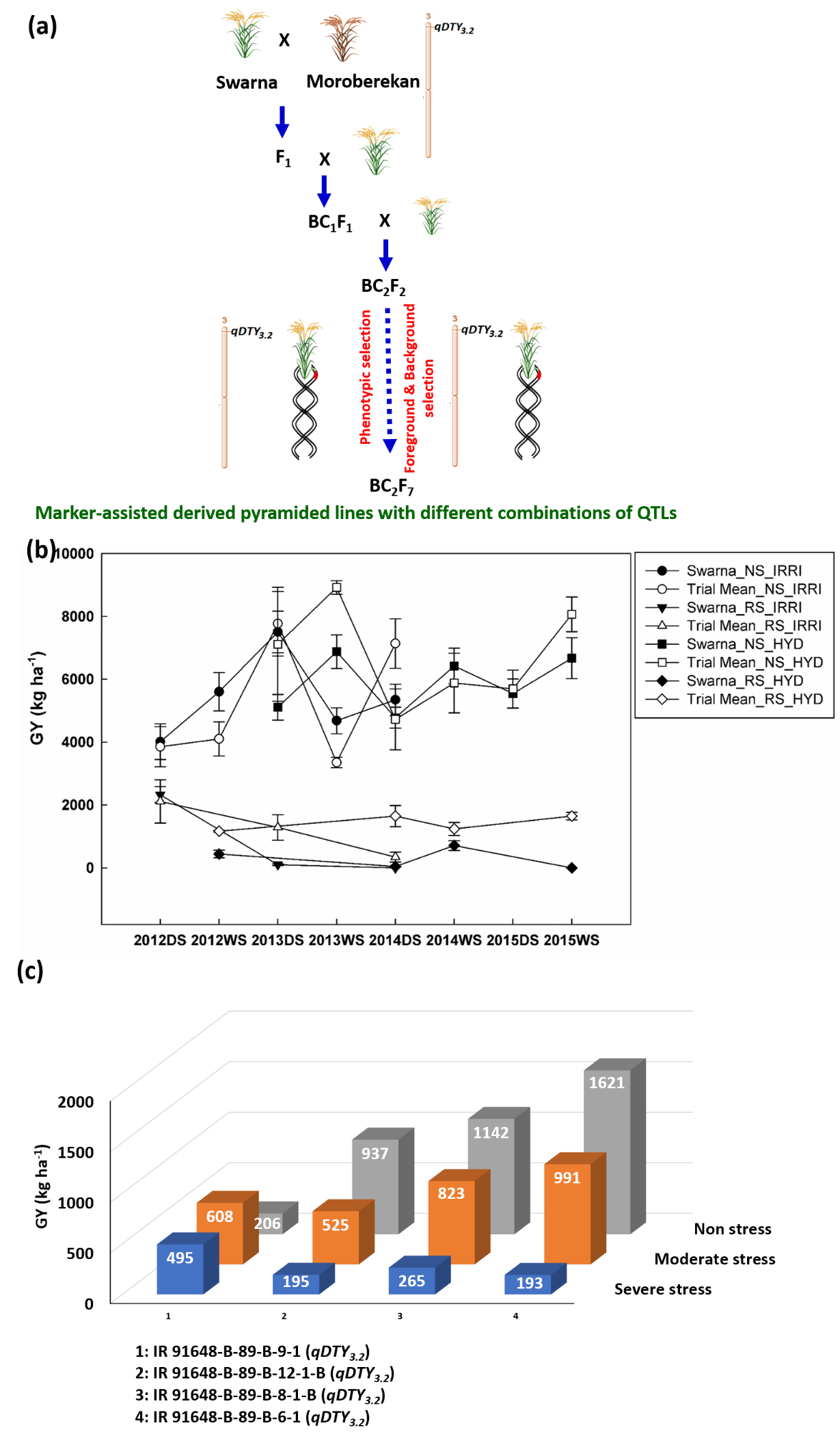


**Figure S8.** **A:** Marker-assisted breeding scheme for the development of pyramided lines in Swarna background. **B:** The mean grain yield performance of introgression lines in comparison with Swarna under NS and RS across seasons at IRRI, Philippines and SAH (Hyderabad, India). **C:** mean grain yield advantage of pyramided lines over Swarna under non-stress, moderate stress and severe stress.


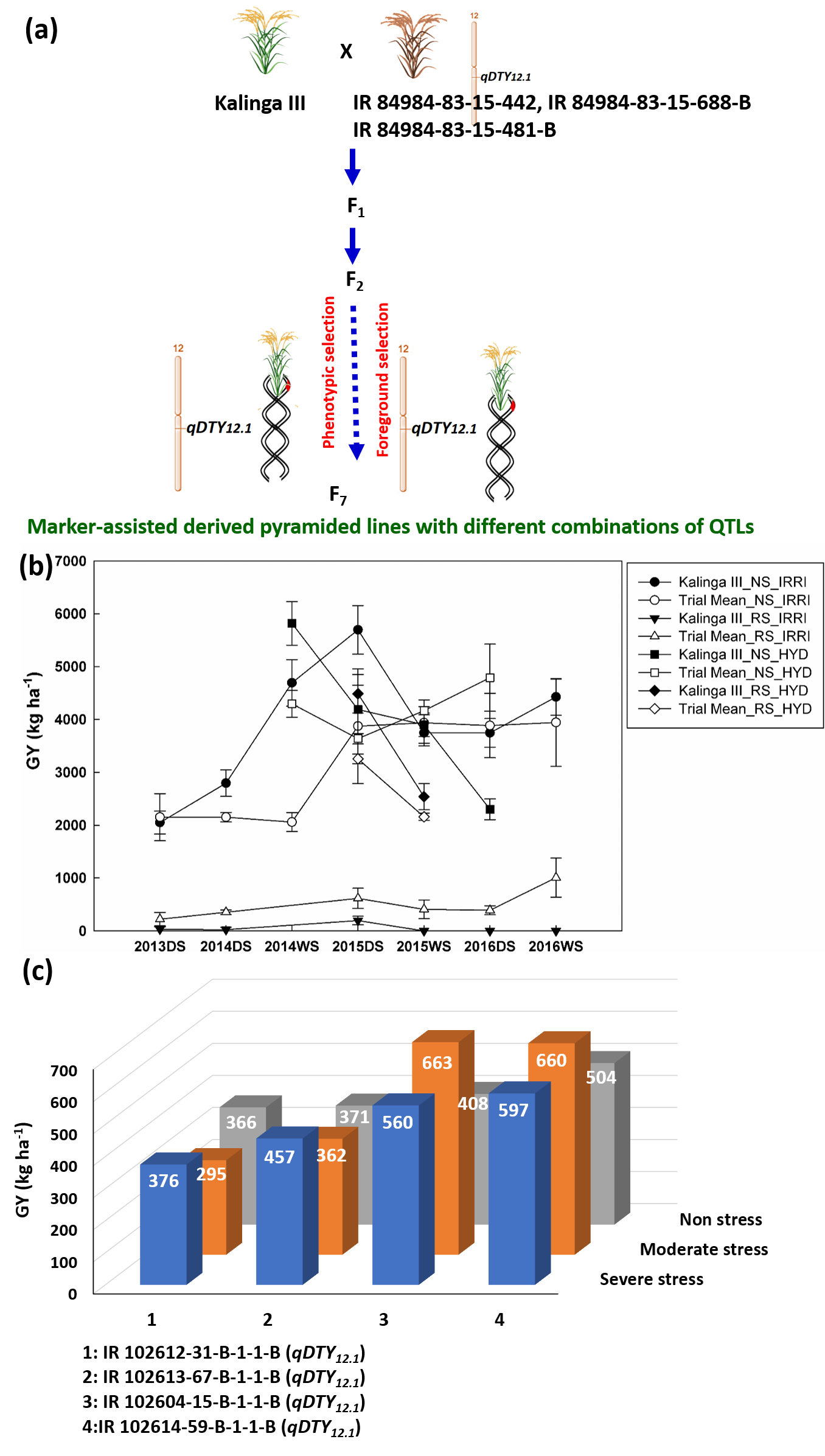


**Figure S9.** **A:** Marker-assisted breeding scheme for the development of pyramided lines in Kalinga III background. **B:** The mean grain yield performance of introgression lines in comparison with Kalinga III under NS and RS across seasons at IRRI, Philippines and SAH (Hyderabad, India). **C:** mean grain yield advantage of pyramided lines over Kalinga III under non-stress, moderate stress and severe stress.


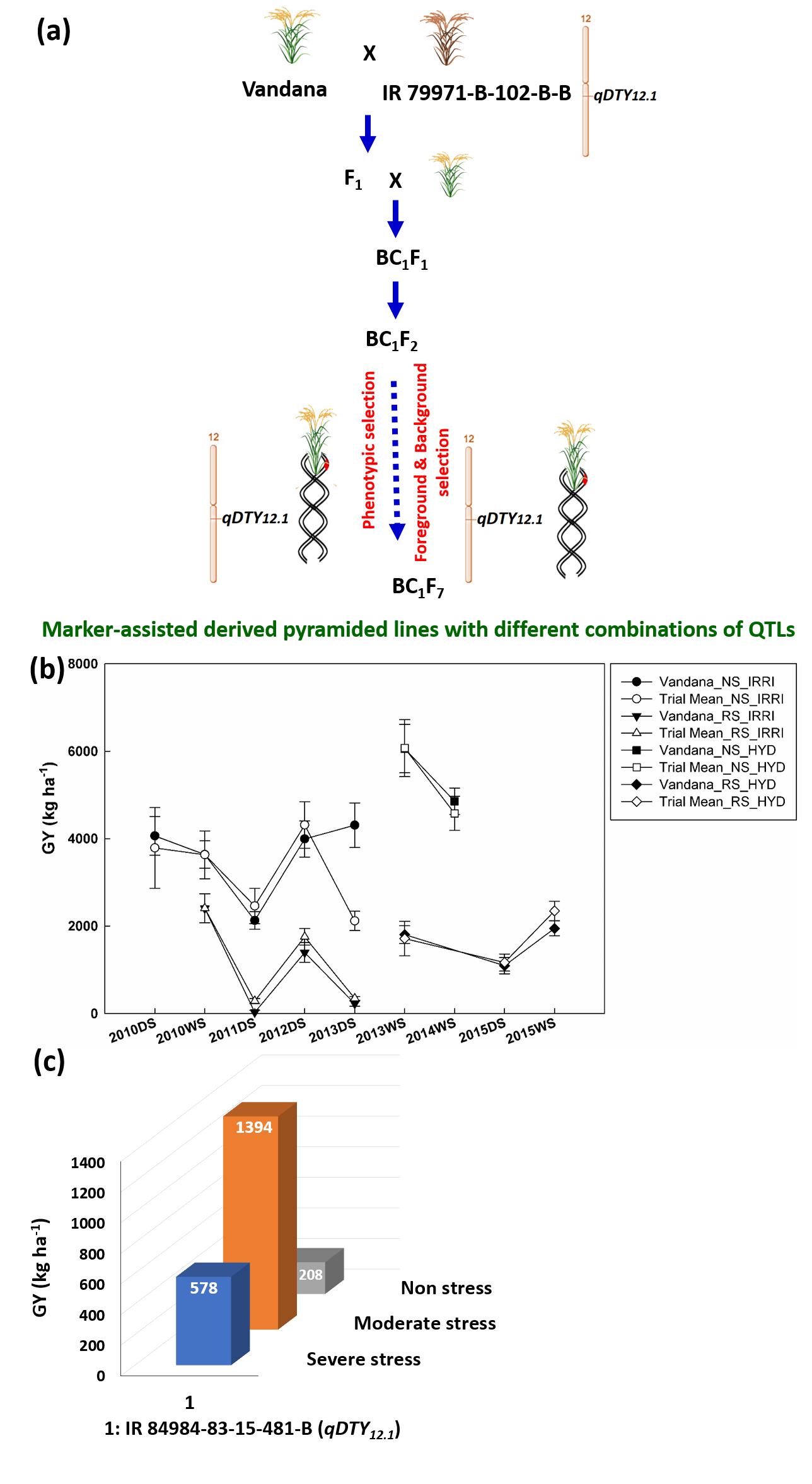


**Figure S10.** **A:** Marker-assisted breeding scheme for the development of pyramided lines in Vandana background. **B:** The mean grain yield performance of introgression lines in comparison with Vandana under NS and RS across seasons at IRRI, Philippines and SAH (Hyderabad, India). **C:** mean grain yield advantage of pyramided lines over Vandana under non-stress, moderate stress and severe stress.


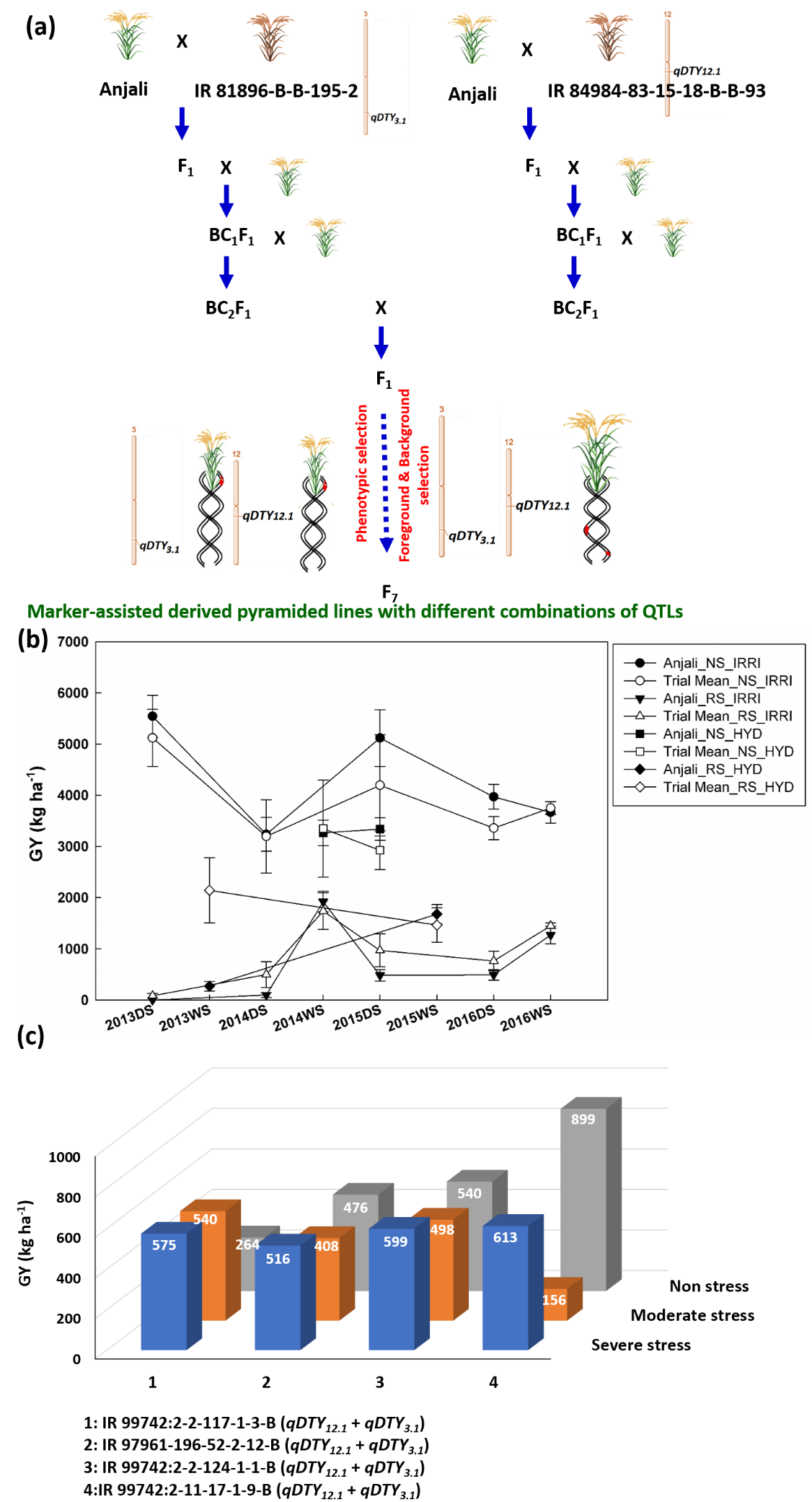


**Figure S11.** **A:** Marker-assisted breeding scheme for the development of pyramided lines in Anjali background. **B:** The mean grain yield performance of introgression lines in comparison with Anjali under NS and RS across seasons at IRRI, Philippines and SAH (Hyderabad, India). **C:** mean grain yield advantage of pyramided lines over Anjali under non-stress, moderate stress and severe stress.
